# Towards the Total Synthesis of Pl-3: Preparation of the Eastern Fragment through a Diastereoselective SmI_2_-Mediated Reformatsky Reaction

**DOI:** 10.1002/ejoc.201300148

**Published:** 2013-03-07

**Authors:** Rita Fürst, Christoph Lentsch, Uwe Rinner

**Affiliations:** [a] Department of Organic Chemistry, University of ViennaWähringer Straße 38, 1090 Vienna, Austria, E-mail: uwe.rinner@univie.ac.at Homepage: http://rinner-group.univie.ac.at

**Keywords:** Natural products, Terpenoids, Samarium, Diastereoselectivity, Multi­drug resistance

## Abstract

The jatrophane diterpene Pl-3, isolated in 2003 from *Euphorbia platyphyllos*, is a structurally complex natural product with highly promising biological properties that include pronounced antiproliferative activity and the inhibition of the efflux-pump activity of multidrug resistance p-glycoprotein. Herein, the synthesis of the eastern fragment of Pl-3 is outlined. The target compound is synthesized in nine synthetic operations in good overall yield, starting from readily available d-ribose. The key step in the preparation of the eastern part of Pl-3 is a diastereoselective SmI_2_-mediated Reformatsky reaction. The proposed route is highly flexible and could also be applied to the synthesis of structurally related jatrophane diterpenes.

## Introduction

The genus *Euphorbia*, a member of the Euphorbiaceae plant family, is one of the largest genera among flowering plants comprising more than 2000 species. *Euphorbia* plants, also known as spurges, are endemic in tropical and subtropical regions as well as in temperate climate zones. Several members are in cultivation and are of great economic importance. For example, the Pará rubber tree (*Hevea brasiliensis*) serves as a source of natural rubber, whereas Cassava (*Manihot esculenta*) is an important annual crop that is rich in carbohydrates. Other members, such as poinsettia (*Euphorbia pulcherrima*), are cultivated for their appealing nature.

Because of their pronounced biological activities, spurges have been common ingredients in traditional herbal folk medicine, and they are mainly used in the treatment of cancerous conditions, swellings, and warts.^1^ The milky sap (latex) is a common attribute of members of the Euphorbiaceae plant family. It contains a large number of structurally diverse diterpenes, which are responsible for the application of spurges in traditional phytotherapy.^2^ Several of these diterpenes were also identified as highly active inhibitors of the adenosine-5′-triphosphate (ATP)-dependent efflux pump p-glycoprotein, which is responsible for multidrug resistance in cancer.^3^ Given that resistance to prevalent drugs is one of the major drawbacks in the development of cancer therapeutics, progress in the synthetic preparation of *Euphorbia* diterpenes is also of medicinal importance.

Chemical interest in the biologically active ingredients of the genus *Euphorbia* started with the isolation of jatrophone by Kupchan in 1970.^4^ Since then, numerous diterpenes of the jatrophane, tigliane, ingenane, and lathyrane frameworks have been isolated and, to some extent, evaluated for their biological activity.^2^ Despite their challenging structural features, only few synthetic efforts towards these fascinating natural products have been reported to date.^5^

**Figure 1:**
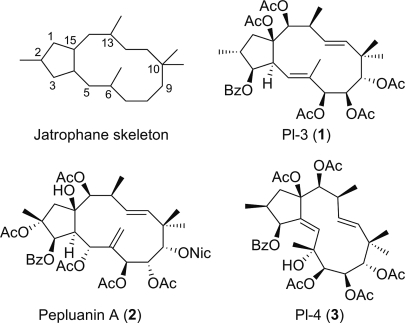
Jatrophane skeleton and representative jatrophane diterpenes.

Pl-3 (**1**) was isolated by Hohmann and co-workers from *Euphorbia platyphyllos* in 2003.^6^ As outlined in Figure 1, jatrophane diterpenes are characterized by a highly functionalized *trans*-bicyclo[10.3.0]pentadecane framework. Individual diterpenes often only differ in the stereochemical pattern of the methyl and hydroxy groups, whereas the substitution pattern remains highly similar.

**Scheme 1:**
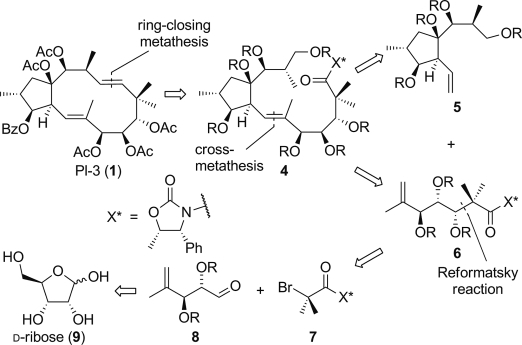
Retrosynthetic analysis.

The retrosynthetic analysis is shown in Scheme 1. The final operation in the synthesis of Pl-3 is a metathesis reaction to close the macrocyclic ring, whereas advanced intermediate **4** should become available from cyclopentane **5** and alkene **6** through cross-metathesis. The key step in the preparation of the eastern part of Pl-3 is a diastereoselective SmI_2_-mediated Reformatsky reaction.^7^ In an attempt to take advantage of the chiral pool, d-ribose was identified as an ideal starting material to access alkene **6**. The utilization of different sugars and the application of an enantiomeric chiral auxiliary in the Reformatsky reaction should furthermore allow the facile preparation of related fragments for the synthesis of other jatrophane diterpenes.

Recently, we reported the synthesis of the cyclopentane moiety of Pl-3 (**1**).5h Herein, we will discuss the preparation of the eastern fragment (i.e., **6**).

## Results and Discussion

**Scheme 2:**
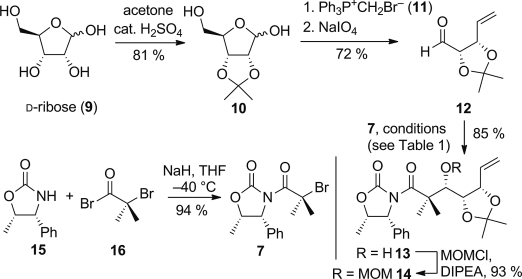
Preparation of alkene **14** (DIPEA = *N*,*N*-diisopropylethylamine).

The first approach towards alkene **6** started with acetonide protection of d-ribose (**9**) as outlined in Scheme 2. Initially, we intended to install the alkene moiety required for the cross-metathesis reaction by Tebbe olefination of the corresponding methyl ketone, which should be accessible from terminal alkene **14** after Wacker oxidation. Thus, acetonide **10** was allowed to react with Wittig salt **11** before periodate cleavage of the formed diol afforded unsaturated aldehyde **12** in excellent yield.^8^

**Scheme 3:**
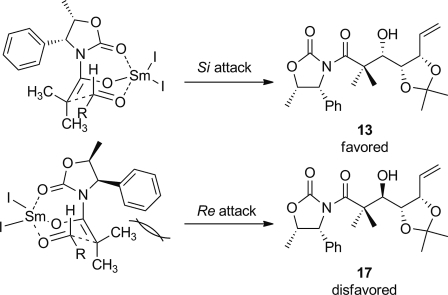
Transition-state model for the diastereoselective Reformatsky reaction.

With **12** in hand, the crucial diastereoselective Reformatsky reaction could be attempted. According to literature precedents, the *S* configuration at the newly generated stereocenter is expected when bromoacyl oxazolidinone **7**, readily available on multigram scale upon acylation of oxazolidinone **15** and 2-bromoisobutyryl bromide (**16**), is allowed to react with aldehyde **12**.7a,^9^ Unfortunately, as shown in Table 1, initial attempts with the use of tin^9^,^10^ and zinc^11^ for the generation of the nucleophile in the Reformatsky reaction resulted in reisolation of the starting material. The desired product, although only in low yield, was first obtained when chromium salts were employed (Table 1, entries 3–6).^12^ Cp_2_TiCl (Cp = cyclopentadienyl) has been described to promote Reformatsky reactions,^13^ but we chose to utilize SmI_2_ next and were able to isolate oxazolidinone **13** in excellent yield as a single diastereomer (the opposite diastereomer was not observed by NMR spectroscopy), which was further converted into methoxymethyl (MOM)-protected intermediate **14**. The high level of selectivity can be explained by the exclusive formation of a pentacoordinate transition state, as described by Thornton and Pridgen.7a,^14^ Steric repulsion of the auxiliary and presumably the bulky geminal dimethyl group efficiently prevents *Re* attack and forces the system to undergo the desired *Si* attack. The *S*-configured hydroxy group in **13** (Scheme 3) is formed through exclusive stereochemical control of the chiral auxiliary.

**Table 1:** Reaction conditions in the diastereoselective Reformatsky reaction

Entry	Metal and solvents	Temp. [°C]	% Yield of13
1	SnCl_2_, LiAlH_4_, THF	0 to r.t.	0
2	Zn, THF	0 to r.t.	0
3	CrCl_2_, LiI, THF	r.t.	7
4	CrCl_2_, THF	r.t.	23
5	CrCl_2_, NiCl_2_, THF	r.t.	<10[a]
6	CrCl_3_, LiAlH_4_, THF	r.t.	<10[a]
7	SmI_2_, THF	–78	85

[a] [a]Yield was determined by analysis of the crude product by ^1^H NMR spectroscopy.

**Scheme 4:**
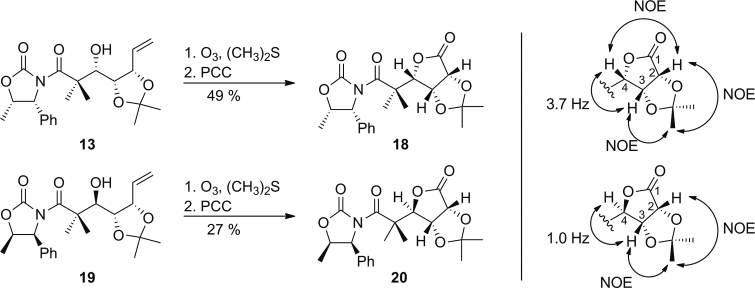
Determination of the stereochemistry.

The proposed configuration of the newly installed hydroxy moiety in **13** was unambiguously confirmed by comparison of the coupling constants between H-4 and H-3 in cyclic intermediates **18** and **20**, according to the Karplus correlation and NOESY experiments (Scheme 4). Cleavage of the terminal double bond in **13** and **19**, available upon Reformatsky reaction of **12** with *ent*-**7**, by ozonolysis was followed by pyridinium chlorochromate (PCC) oxidation of the resulting lactol. Whereas the H-4–H-3 coupling constant in **20**, which originated from the alcohol with the *R* configuration, was 1.0 Hz, a value of 3.7 Hz was measured for cyclic intermediate **18**, and thus, the stereochemical configuration of alcohol **13** could be confirmed.

**Scheme 5:**
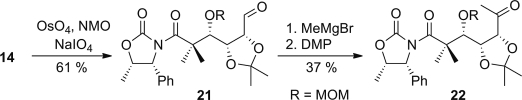
Preparation of methyl ketone **22** (NMO = *N*-methylmorpholine-*N*-oxide; DMP = Dess–Martin periodinane).

All attempts to convert alkene **14** into methyl ketone **22** through Wacker oxidation failed and only resulted in reisolation of the starting material. As outlined in Scheme 5, dihydroxylation of the terminal double bond with OsO_4_ and periodate cleavage of the resulting diol allowed the isolation of highly unstable aldehyde **21**. However, the conversion of **21** into methyl ketone **22** proved to be troublesome, as addition of the methyl Grignard reagent and subsequent oxidation of the secondary alcohol delivered the desired compound in low and irreproducible yield.

**Scheme 6:**
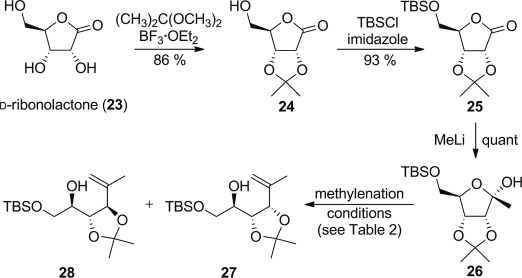
Second approach – preparation of alkenes **27** and **28** (TBS = *tert*-butyldimethylsilyl).

In a modified approach, we intended to install the methyl ketone at an early stage to circumvent the problems discussed above. As shown in Scheme 6, the route started with Lewis acid promoted conversion of d-ribonolactone (**23**) into acetonide **24**. Silylation of the primary hydroxy group delivered lactone **25**, which was treated with methyllithium to afford lactol **26** as a masked methyl ketone in quantitative yield as a single diastereomer.^15^ Next, we intended to install the terminal double bond. Methylenation of **26** with Tebbe's reagent was unsuccessful, and even at elevated temperature no conversion could be detected (Table 2, entries 1 and 2). Surprisingly, when allowed to react with the methyl Wittig reagent (**11**) under standard reaction conditions in THF with *t*BuOK as the base, α-racemization occurred and a diastereomeric mixture of **27**/**28** in a 5:1 ratio was isolated in moderate 34 % yield. When toluene was used as the solvent, the exclusive formation of desired alkene **27** was observed; however, the isolated yield did not exceed 30 %. Several other protocols were investigated: potassium hexamethyldisilazane (KHMDS) in THF afforded exclusively undesired diastereomer **28** (Table 2, entry 7), whereas other bases such as NaH and BuLi resulted in reisolation of the starting material. As the yield in the methylenation reaction could not be further improved, another approach was elaborated.

**Table 2:** Methylenation of lactol 26

Entry	Reagent/solvent	Temp.	Time	% Yield
		[°C]	[h]	(27/28)
1	Tebbe reagent/THF	0	3	0
2	Tebbe reagent/THF	r.t. to 80	3	0
3	*t*BuOK/THF[a]	0 to r.t.	4	34 (5:1)
4	NaH/DMSO[b]	r.t. to 50	12	0
5	NaH/THF[c]	–20 to r.t.	12	0
6	*n*BuLi/THF[d]	–78 to r.t.	12	0
7	KHMDS/THF[a]	–78 to r.t.	14	13 (<1:99)
8	*t*BuOK/toluene[a]	–78 to r.t.	4	30 (>99:1)

[a] [a]The Wittig salt was deprotonated at 0 °C over 20 min and at r.t. over 1.5 h before **26** was added.

[b] NaH was heated in DMSO to 75 °C for 45 min before the Wittig salt was added at 0 °C, and deprotonation was accomplished at r.t. over 20 min.

[c] The Wittig salt was deprotonated at r.t. over 2 h.

[d] The Wittig salt was deprotonated at –78 °C over 1 h.

**Scheme 7:**
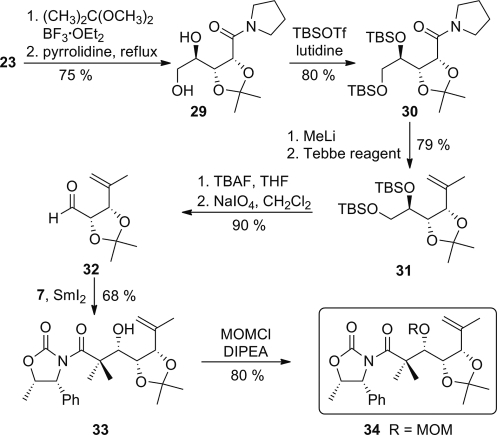
Preparation of alkene **34** (TBAF = tetrabutylammonium fluoride).

In our third approach, protection of d-ribonolactone (**23**) as its acetonide was followed by exposure of the lactone to pyrrolidine at elevated temperature to obtain amide **29** in excellent yield (Scheme 7). Next, silylation of both hydroxy functionalities was followed by addition of methyllithium to deliver the corresponding methyl ketone, which could be smoothly converted into alkene **31** upon reaction with Tebbe's reagent. Removal of both silyl ethers and subsequent oxidative cleavage of the vicinal diol with NaIO_4_ delivered aldehyde **32**, the precursor for the crucial diastereoselective Reformatsky reaction. Again, as discussed for the first approach, the desired secondary alcohol was obtained in good yield as a single isomer when a degassed, precooled THF solution of bromide **7** and aldehyde **32** was added to a SmI_2_ solution at –78 °C. Final MOM protection delivered advanced intermediate **34** and completed the preparation of the eastern part of Pl-3 (**1**).

## Conclusions

We have established a general nine-step sequence for the synthesis of the eastern fragment of Pl-3. The Reformatsky reaction, described in detail within this manuscript, can be employed in the synthesis of jatrophane diterpenes to establish recurring structural motifs within the eastern part. The application of different carbohydrate derivatives as the starting material in combination with the highly diastereoselective SmI_2_-mediated Reformatsky reaction grants access to a variety of structurally related Euphorbiaceae diterpenes. Owing to the diastereoselective control of the chiral auxiliary within the SmI_2_-mediated Reformatsky reaction, the utilization of this reaction will be of interest for other synthetic applications. The scope of the synthetic route and the completion of the preparation of Pl-3 are currently under investigation in our group.

## Experimental Section

**Preparation of 33:** To a solution of SmI_2_ (0.1 m in THF, 100 mL, 10 mmol, 2.5 equiv.) in a 250 mL round bottom Schlenk flask was added a solution of bromide **7** (1.44 g, 4.41 mmol, 1.1 equiv.) and aldehyde **32** (683 mg, 4.01 mmol, 1.0 equiv.) in degassed THF (60 mL, 3 pump–freeze–thaw cycles) at –78 °C by cannula. The reaction mixture was stirred for 1 h at –78 °C before it was quenched by the addition of aqueous saturated solutions of sodium thiosulfate (50 mL) and sodium hydrogen carbonate (50 mL) at –78 °C, and the biphasic system was warmed to room temperature. The two phases were separated, and the aqueous layer was extracted with ethyl acetate (3×). The combined organic extract was dried with sodium sulfate and filtered, and the organic solvents were removed under reduced pressure to deliver alcohol **33** as a light-yellow oil, which was further purified by flash column chromatography (hexanes/ethyl acetate, 9:1) to provide **33** (1.14 g, 68 %).

**Supporting Information** (see footnote on the first page of this article): Experimental details and copies of the ^1^H NMR and ^13^C NMR spectra of all compounds described in this communication.

## Supplementary material

As a service to our authors and readers, this journal provides supporting information supplied by the authors. Such materials are peer reviewed and may be re-organized for online delivery, but are not copy-edited or typeset. Technical support issues arising from supporting information (other than missing files) should be addressed to the authors.
