# Supplementary material for: Towards the Total Synthesis of Pl-3: Preparation of the Eastern Fragment through a Diastereoselective SmI2-Mediated Reformatsky Reaction
Source: European J Org Chem. 2013 Mar 7;2013(12):2293–7. doi: 10.1002/ejoc.201300148 (PMC3654569; doi:10.1002/ejoc.201300148)
Supplement: Supplementary file 1 [file ejoc2013-2293-SD1.pdf]

Eur. J. Org. Chem. **2013** · © WILEY-VCH Verlag GmbH & Co. KGaA, 69451 Weinheim, 2013 · ISSN 1099–0690

SUPPORTING INFORMATION

DOI: 10.1002/ejoc.201300148
Title: Towards the Total Synthesis of Pl-3: Preparation of the Eastern Fragment through a Diastereoselective SmI_2-Mediated Reformatsky Reaction
Author(s): Rita Fürst, Christoph Lentsch, Uwe Rinner*

Table of Contents

General methods 2

Experimental part 3

References 15

NMR Spectra 16

General methods

Synthetic methods: All non-aqueous reactions were carried out under a positive pressure of argon using oven-dried (100 °C) or flame-dried glassware (under vacuum) unless noted otherwise.

Solvents and chemical purification: THF was dried by distillation from potassium under argon. Diethyl ether, dimethoxyethane, benzene and toluene were purified by distillation and dried by distillation from sodium/benzophenone ketyl under argon. DMSO and *N*,*N*-dimethylformamide were dried by distillation from calcium hydride under reduced pressure. DCM was purified by distillation and dried by distillation from phosphor pentoxide and passage over aluminum oxide, neutral, activity. Dry solvents were stored under an argon atmosphere over molecular sieves (4 Å).

Triethylamine, diethylisopropylamine and diisopropylamine were distilled from calcium hydride under an atmosphere of argon prior to use.

All other commercially available reagents were used without further purification. Except if indicated otherwise, reactions were magnetically stirred and monitored by thin layer chromatography using Merck silica gel 60-F254 glass plates. The plates were developed with a mixture of hexane/ethyl acetate or toluene/ethyl acetate. Unless the compound was colored, UV-active spots were detected at longwave UV (254 nm) or shortwave (180 nm). Most plates were additionally treated with one of the following visualization reagents: CAM [H_2SO_4 (conc., 22 mL), phosphormolybdic acid (20 g), $Ce(SO_4)_2$ (0.5 g), 378 mL H_2O)] or silica gel impregnated with iodine.

Chromatography: Preparative column chromatography and flash column chromatography were performed with silica gel 60 from Merck (0.040-0.063 µm, 240-400 mesh).

For HPLC separations on analytical scale module systems from Jasco (PU-980, UV- 975 detector, RI-930 RI detector, 250 x 4 mm column) were used. The adsorbent was Superphere Si 60 (40 µm, Merck) or Nucleosil 50 (4 µm, Macherey-Nagel). The semipreparative and preparative scale was covered by module systems from Dynamax (SD-1 pump, UV-1 UV detector), Knauer (RI detector) and Shimadzu (LC-8A, SPD-20A UV/VIS Detector, LC-20AT Bus Module).

Solvents were removed by rotary evaporation at 30 °C at the appropriate pressure, unless stated otherwise. Yields refer to chromatographically purified and spectroscopically pure compounds, unless stated otherwise.

Optical rotations: Optical rotations were measured at the sodium D line with a 100 mm path length cell, and are reported as follows: $[\alpha]^{T}_{D}$, concentration (g/100 mL), and solvent.

NMR spectra: NMR spectra were recorded either on a Bruker Avance AV 400, DRX 400, or DRX 600 MHz spectrometer. Unless stated otherwise, all NMR spectra were measured in $CDCl_3$ solutions and referenced to the residual $CDCl_3$ signal (^{1}H, δ = 7.26, ^{13}C, δ = 77.16). All ^{1}H and ^{13}C shifts are given in ppm (s = singlet, d = doublet, dd = doublet of doublets, t = triplet, q = quartet, quint = quintet, m = multiplet, br = broadened signal). Coupling constants *J* are given in Hz. Assignments of proton resonances were confirmed, when possible, by correlated spectroscopy (COSY, HSQC, HMBC, TOCSY, NOESY).

IR spectra: IR spectra were recorded using a Perkin-Elmer 1600 Series FTIR spectrometer and are reported in wave numbers (cm^{-1}). All compounds were measured as a thin film on silicon single crystal plate.

Experimental part

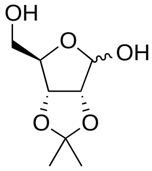

10

(3a*R*,6*R*,6a*R*)-6-(Hydroxymethyl)-2,2-dimethyltetrahydrofuro[3,4-d][1,3]dioxol-4-ol (10). To a suspension of D-ribose (5 g, 33.3 mmol, 1 eq) in acetone (62.5 mL) was added dropwise a catalytic amount of concentrated sulfuric acid (150 µL, 0.1 eq) at room temperature. The reaction mixture was stirred for twelve hours at ambient temperature before it was neutralized with solid sodium bicarbonate. The suspension was stirred for additional five hours before the precipitate was removed by filtration and the solvent was evaporated under reduced pressure. The crude product was purified by flash column chromatography (hexanes/ethyl acetate 2:1) providing lactole **10** (5.2 g, 81%) as colorless oil.

^{1}H NMR (400 MHz, $CDCl_3$): δ = 1.32 (s, 3H), 1.49 (s, 3H), 3.47 (bs, 1H), 3.62-3.83 (m, 2H), 4.40 (bs, 1H), 4.58 (d, *J* = 5.83 Hz, 1H), 4.84 (d, *J* = 5.83 Hz, 1H), 5.42 (bs, 1H) ppm.

^{13}C NMR (100 MHz, $CDCl_3$): δ =24.9 (CH_3), 26.5 (CH_3), 63.9 (CH_2), 81.9 (CH), 87.0 (CH), 88.0 (CH), 103.2 (CH), 112.3 (C) ppm.

These spectral characteristics are identical to those previously reported.[1]

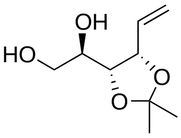

S1

(*R*)-1-((4*R*,5*S*)-2,2-Dimethyl-5-vinyl-1,3-dioxolan-4-yl)ethane-1,2-diol (S1). To a stirred suspension of methyltriphenylphosphonium bromide (3.5 g, 9.8 mmol, 3.5 eq) in THF (10 mL) was added potassium *tert*-butoxide (1.1 g, 9.8 mmol, 3.5 eq) at 0 °C. The reaction mixture was kept at that temperature for 20 min before it was warmed to room temperature and stirred for one additional hour. After recooling to 0 °C, lactole **10** (530 mg, 2.8 mmol, 1.0 eq) was dissolved in 2.5 mL THF and added *via* syringe. The resulting yellow mixture was stirred for 14 hours at room temperature. The reaction was then quenched by the addition of saturated ammonium chloride solution. The aqueous phase was extracted three times with ethyl acetate, the combined organic extracts were dried over solid sodium sulfate and the solvent was removed under reduced pressure. Afterwards, the crude material was purified by flash column chromatography (hexanes/ethyl acetate 1:2) affording the Wittig-product (**S1**, 470 mg, 89%) as light yellow oil.[2]

^{1}H NMR (400 MHz, $CDCl_3$): δ = 1.37 (s, 3H), 1.48 (s, 3H, CH_3), 2.0-2.08 (bs, 1H), 2.19-2.42 (m, 1H), 3.67-3.87 (m, 3H), 4.11 (dd, *J* = 6.6, 8.4 Hz, 1H), 4.71 (bt, *J* = 6.58 Hz, 1H), 5.33 (dt, *J* = 1.2, 10.4 Hz, 1H), 5.47 (dt, *J* = 1.2, 17.3 Hz, 1H), 6.01 (ddd, *J* = 6.6, 10.4, 17.3 Hz, 1H) ppm.

^{13}C NMR (100 MHz, $CDCl_3$): δ = 25.4 (CH_3), 27.9 (CH_3), 64.5 (CH_2), 70.0 (CH), 78.4 (CH), 78.7 (CH), 109.2 (C), 118.8 (CH_2), 133.9 (CH) ppm.

IR (thin film) ν 3384, 2987, 2937, 1372, 1216, 1055, 928, 872, 798 cm^{-1}.

HRMS (EI) calcd for $C_9H_{16}O_4$ $[M+Na]^+$, 211.0947; found 211.0940 +/− 5ppm.

Optical Rotation: $[\alpha]^{20}_{D}$ (c 1.0, $CHCl_3$) = +25.9°.

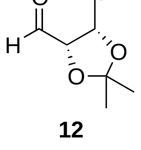

12

(4*S*,5*S*)-2,2-Dimethyl-5-vinyl-1,3-dioxolane-4-carbaldehyde (12). Diol **S1** (3.3 g, 17.5 mmol, 1.0 eq) was dissolved in methylene chloride (60 mL) before sodium periodate (5.6 g, 26.3 mmol, 1.5 eq) dissolved in 40 mL water was added dropwise *via* syringe. The cooling bath was removed and the

reaction mixture was stirred at room temperature for two hours before it was diluted with water. The layers were separated and the aqueous phase was extracted three times with methylene chloride. The combined organic extracts were dried over sodium sulfate, filtered and the organic solvent was removed in vacuum (180-250 mbar, 30 °C water bath temperature). The resulting very labile product was filtered through a short plug of silica gel (pentanes/diethyl ether 3:1) delivering aldehyde **12** (2.2 g, 81%) as colorless oil which was immediately used for the next step.

^{1}H NMR (400 MHz, $CDCl_3$): δ = 1.44 (s, 3H), 1.62 (s, 3H), 4.41 (dd, *J* = 3.0, 7.5 Hz, 1H), 4.86 (bt, *J* = 7.5 Hz, 1H), 5.33 (dt, *J* = 1.3, 10.5 Hz, 1H), 5.47 (dt, *J* = 1.3, 17.1 Hz, 1H), 5.74 (ddd, *J* = 6.8, 10.3, 17.3 Hz, 1H), 9.56 (d, *J* = 3.0 Hz, 1H) ppm.

These spectral characteristics are identical to those previously reported.[1a]

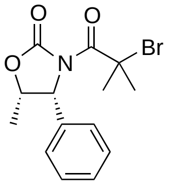

7

(4*R*,5*S*)-3-(2-Bromo-2-methylpropanoyl)-5-methyl-4-phenyloxazolidin-2-one (7**).** To a solution of (4*R*,5*S*)-5-methyl-4-phenyloxazolidin-2-one (3.0 g, 16.9 mmol, 1.0 eq) in dry THF (30 mL) was added sodium hydride (608 mg, 23.4 mmol, 1.5 eq) in one portion at room temperature. After the addition, the resulting suspension was cooled to −40 °C and a solution of 2-bromoisobutyryl bromide in 80 mL THF was added. The reaction mixture was stirred for one hour at −40 °C and one additional hour at 0 °C before TLC control showed total consumption of the starting material. The reaction was terminated by addition of saturated ammonium chloride solution. The two phases were separated and the aqueous layer was extracted three times with ethyl acetate. The combined organic extracts were dried over solid sodium sulfate and afterwards the solvent was removed under reduced pressure. The crude product was purified by flash column chromatography (hexanes/ethyl acetate 5:1) giving bromide **7** (5.15 g) in 94% yield.

^{1}H NMR (400 MHz, $CDCl_3$): δ = 0.94 (d, *J* = 6.6 Hz, 3H), 2.10 (s, 3H), 2.15 (s, 3H), 4.81 (quint, *J* = 6.7 Hz, 1H), 5.73 (d, *J* = 7.1 Hz, 1H), 7.29-7.46 (m, 5H) ppm.

^{13}C NMR (100 MHz, $CDCl_3$): δ = 14.2 (CH_3), 30.6 (CH_3), 31.7 (CH_3), 57.1 (C), 57.6 (CH), 79.1 (CH), 125.8 (CH) 128.9 (CH), 129.0 (CH), 133.6 (C), 151.2 (C), 171.57 (C) ppm.

IR (thin film) ν 1788, 1682, 1455, 1339, 1284, 1191, 1122, 1068, 966, 699 cm^{-1}.

HRMS (EI) calcd for $C_{14}H_{16}BrNO_3$ $[M+Na]^+$, 325.0314; found 325.0318 +/− 5ppm.

Optical Rotation: $[\alpha]^{20}_D$ (c 0.1, $CHCl_3$) = +28.4°.

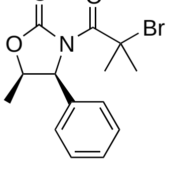

S2

(4*S*,5*R*)-3-(2-Bromo-2-methylpropanoyl)-5-methyl-4-phenyloxazolidin-2-one (S2**).** Bromide **S2** was synthesized following the same procedure as described for bromide **7**, starting from (4*S*,5*R*)-5-methyl-4-phenyloxazolidin-2-one (3 g, 23.4 mmol) in 94% yield.

Optical Rotation: $[\alpha]^{20}_D$ (c 0.1, $CHCl_3$) = −28.4°.

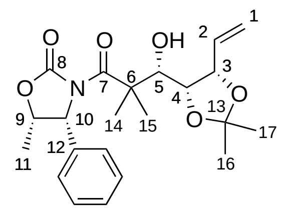

13

(4*R*,5*S*)-3-((*S*)-3-((4*R*,5*S*)-2,2-Dimethyl-5-vinyl-1,3-dioxolan-4-yl)-3-hydroxy-2,2-dimethylpropanoyl)-5-methyl-4-phenyloxazolidin-2-one (13).

SmI_2 methode: A solution of SmI_2 (100 mL, 0.1 M in THF, 10.0 mmol, 2.5 eq) was cannulated in a 250 mL round bottom Schlenk flask which was precooled to -78 °C. A solution of bromide **7** (1.44 g, 4.4 mmol, 1.1 eq) and aldehyde **12** (624 mg, 4 mmol, 1.0 eq) in 60 mL degassed THF (3 pump freeze thaw cycles) was added to the SmI_2 solution *via* cannula. The reaction mixture was stirred for one hour at −78 °C before the reaction was quenched by the addition of aqueous saturated solutions of sodium thiosulfate (50 mL) and sodium bicarbonate (50 mL) at −78 °C and the biphasic mixture was allowed to warm to room temperature. The two phases were separated, and the aqueous layer was extracted three times with ethyl acetate. The combined organic extracts were dried over sodium sulfate, filtered and the organic solvents were removed under reduced pressure delivering alcohol **13** as light oil which was further purified by flash column chromatography (hexanes/ethyl acetate 9:1 to 5:1) in 85% yield (1.37 g).

Chromium method I: Chromium dichloride (157 mg, 128 mmol, 4.0 eq) and lithium chloride (21 mg, 0.16 mmol, 0.5 eq) were suspended in freshly distilled THF (2 mL) and vigorously stirred at room temperature. Aldehyde **12** was added, followed by bromide **7,** each dissolved in THF (1.0 mL). The reaction mixture was stirred for 1.5 h at room temperature, 1 h 20 min at 40 °C and 3 h at 60 °C before it was quenched with brine. The layers were separated and the aqueous phase was extracted three times with ethyl acetate. The combined organic extracts were dried over sodium sulfate, filtered and the solvent was removed *in vacuo*. The resulting crude product was purified by flash column chromatography (hexanes/ethyl acetate 9/1) delivering alcohol **13** (8.5 mg) in 7% yield.

Chromium method II: Chromium dichloride (98 mg, 0.8 mmol, 2.5 eq) was suspended in freshly distilled THF (1.5 mL). Aldehyde **12** (50 mg, 0.32 mmol, 1.0 eq) and bromide **7** (114 mg, 0.35 mmol, 1.1 eq), dissolved in 1.0 mL THF each, were added consecutively within five minutes at room temperature. The resulting mixture was stirred for seven hours at room temperature before the reaction was quenched with brine. The two layers were separated and the aqueous phase was extracted three times with ethyl acetate. The combined organic extracts were dried over sodium sulfate, filtered and the solvent was removed *in vacuo*. The resulting crude product was purified by flash column chromatography (hexanes/ethyl acetate 9:1) delivering alcohol **13** (30 mg) in 23% yield.

Chromium method III: Chromium trichloride (136 mg, 0.86 mmol, 2.7 eq) was suspended in 1.3 mL THF and a solution of lithium aluminum hydride (4.0 M in diethyl ether, 0.113 mL, 0.45 mmol, 1.4 eq) was added at 0 °C under vigorous stirring. The resulting black suspension was stirred for 45 min at 0 °C before aldehyde **12** (50 mg, 0.32 mmol, 1.0 eq) was added, followed by the addition of bromide **7** (147 mg, 0.45 mmol, 1.4 eq) at 0 °C; both dissolved in 0.5 mL THF, respectively. The reaction mixture was allowed to warm to room temperature over a period of 3.5 hours. The reaction was terminated by the addition of brine, the two layers were separated and the aqueous phase was extracted three times with ethyl acetate. The combined organic extracts were dried over sodium sulfate, filtered and the organic solvents were removed under reduced pressure. The crude product was purified by flash column chromatography (hexanes/ethyl acetate 9/1) delivering alcohol **13** (12.5 mg, 10%) as light yellow oil.

^{1}H NMR (400 MHz, $CDCl_3$): δ = 0.92 (d, *J* = 6.6 Hz, 3H, CH_3-11), 1.35 (s, 3H, CH_3-14 or 15), 1.36 (s, 3H, CH_3-16 or 17), 1.44 (s, 3H, CH_3-14 or 15), 1.51 (s, 3H, CH_3-16 or 17), 3.44 (d, *J* = 10.1 Hz, 1H, OH), 4.28 (d, *J* = 7.6 Hz, 1H, H-4), 4.35 (d, *J* = 10.1 Hz, 1H, H-5), 4.67 (dd, *J* = 7.6, 8.0 Hz, 1H, H-3), 4.78 (quint, *J* = 6.6 Hz, 1H, H-9), 5.33-5.42 (m, 2H, H-1a,b), 5.66 (d, *J* = 6.8, 1H, H-10), 6.14 (ddd, *J* = 8.1, 10.1, 17.5 Hz, 1H, H-2), 7.28-7.33 (m, 2H, CH-phenyl), 7.34-7.45 (m, 3H, CH-phenyl) ppm.

^{13}C NMR (100 MHz, $CDCl_3$): δ = 14.0 (CH_3-11), 20.0 (CH_3-14 or 15), 23.3 (CH_3-14 or 15), 24.9 (CH_3-16 or 17), 27.0 (CH_3-16 or 17), 50.7 (C-6), 57.8 (CH-9), 71.5 (CH-5), 76.1 (CH-4), 79.3 (CH-10), 80.4 (CH-3), 108.8 (C-13), 119.8 (CH_2-1), 125.8 (CH-phenyl), 128.8 (CH-phenyl), 128.9 (CH-phenyl), 133.6 (C-12), 152.7 (C-8), 177.0 (C-7) ppm.
IR (thin film) ν 3424, 2987, 2937, 1773, 1687, 1456, 1341, 1255, 1150, 1119, 1045, 939, 889, 768, 701, 657 cm^{-1}.
HRMS (EI) calcd for $C_{22}H_{29}NO_6$ $[M+Na]^+$, 426.1893; found 426.1890 +/− 5ppm.
Optical Rotation: $[\alpha]^{20}_D$ (c 1.0, $CHCl_3$) = +52.8°.

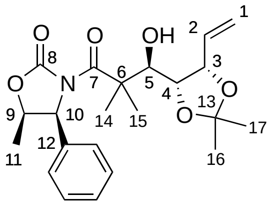

19

(4*R*,5S)-3-((*R*)-3-((4*R*,5*S*)-2,2-Dimethyl-5-vinyl-1,3-dioxolan-4-yl)-3-hydroxy-2,2-dimethylpropanoyl)-5-methyl-4-phenyloxazolidin-2-one (19).
Diastereomer **19** was prepared following the same procedures as described above for alcohol **13**.

	Aldehyde (**12**)	Bromide (**S2**)	Yield (**19**)
SmI_2 (4.9 eq)	50 mg, 0.32 mmol, 1.0 eq	111 mg, 0.34 mmol, 1.06 eq	56 mg (43%)
$CrCl_2$ (2.5 eq), LiI (0.1 eq)	50 mg, 0.32 mmol, 1.0 eq	115 mg, 0.35 mmol, 1.1 eq	50 mg (39%)

^{1}H NMR (400 MHz, $CDCl_3$): δ = 0.93 (d, *J* = 6.6 Hz, 3H, CH_3-11), 1.35 (s, 3H, CH_3-16 or 17), 1.38 (s, 3H, CH_3-14 or 15), 1.48 (s, 3H, CH_3-16 or 17), 1.49 (s, 3H, CH_3-14 or 15), 2.21 (d, *J* = 6.1 Hz, 1H, OH), 4.18 (dd, *J* = 6.3, 9.9 Hz, 1H, H-4), 4.64 (dd, *J* = 6.3, 7.5 Hz, 1H, H-3), 4.70-4.80 (m, 2H, H-5, H-9), 5.29-5.42 (m, 2H, H-1a,b), 5.62 (d, *J* = 7.1 Hz, 1H, H-10), 5.99 (ddd, *J* = 7.5, 10.2, 17.1 Hz, 1H, H-2), 7.27-7.32 (m, 2H, CH-phenyl), 7.33-7.44 (m, 3H, CH-phenyl) ppm.
^{13}C NMR (100 MHz, $CDCl_3$): δ = 14.6 (CH_3-11), 20.0 (CH_3- 14 or 15), 20.8 (CH_3-14 or 15), 25.2 (CH_3-16 or 17), 28.0 (CH_3-16 or 17), 49.6 (C-6), 57.6 (CH-9), 69.5 (CH-5), 77.9 (CH-4), 79.2 (CH-10), 79.8 (CH-3), 109.1 (C-13), 118.9 (CH_2-1), 125.7 (CH-phenyl), 128.8 (CH-phenyl), 133.6 (C-12), 135.0 (CH-2), 152.5 (C-8), 175.9 (C-7) ppm.
IR (thin film) ν 3424, 2987, 2937, 1773, 1687, 1456, 1341, 1255, 1150, 1119, 1045, 939, 889, 768, 701, 657 cm^{-1}.
HRMS (EI) calcd for $C_{22}H_{29}NO_6$ $[M+Na]^+$, 426.1893; found 426.1890 +/− 5ppm.
Optical Rotation: $[\alpha]^{20}_D$ (c 1.0, $CHCl_3$) = +8.3°.

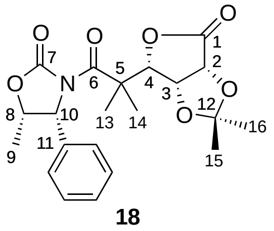

18

(4*R*,5*S*)-3-(2-((3a*R*,4*S*,6a*R*)-2,2-Dimethyl-6-oxotetrahydrofuro[3,4-d][1,3]dioxol-4-yl)-2-methylpropanoyl)-5-methyl-4-phenyloxazolidin-2-one (18). Alkene **13** (58 mg, 0.14 mmol, 1.0 eq) was dissolved in 5 mL methylene chloride and cooled to −78 °C. A stream of ozone was bubbled through the reaction mixture (for approximately 2 min) until the solution turned characteristically blue. In order to remove excess of ozone from the reaction mixture, oxygen was bubbled through the solution and the reaction mixture turned colorless. Next, the solution was purged with argon for approximately two minutes before the reaction mixture was allowed to warm to room temperature over a period of 14

hours. The solvent was removed under reduced pressure delivering an inseparable mixture of diastereomers of the corresponding lactol (43 mg, 75%) as colorless oil, which was used for the next step without any purification.

To a solution of the crude mixture of lactols from above (20 mg, 0.05 mmol, 1.0 eq) in methylene chloride (1.0 mL) was added sodium acetate (8 mg, 0.1 mmol, 2.0 eq) and PCC (22 mg, 0.1 mmol, 2.0 eq) at room temperature. The reaction mixture was stirred at room temperature for twelve hours. The resulting suspension was filtered through a short plug of silica gel and the solvent was removed under reduced pressure delivering the crude lactone as yellow oil. Further purification by flash column chromatography (hexanes/ethyl acetate 9:1 to 5:1) delivered lactone **18** (13 mg) in 65% yield.

^{1}H NMR (400 MHz, $CDCl_3$): δ = 0.91 (d, *J* = 6.6 Hz, 3H, CH_3-9), 1.35 (s, 3H, CH_3-15), 1.47 (s, 3H, CH_3-16), 1.60 (s, 3H, CH_3-13 or 14), 1.65 (s, 3H, CH_3-13 or 14), 4.82 (d, *J* = 5.6 Hz, 1H, H-2), 4.83 (quint, *J* = 4.8 Hz, 1H, H-8) 4.99 (dd, *J* = 3.7, 5.5 Hz, 1H, H-3), 5.24 (d, *J* = 3.7 Hz, 1H, H-4), 5.68 (d, *J* = 7.3 Hz, 1H, H-10), 7.28-7.32 (m, 2H, CH-phenyl), 7.36-7.46 (m, 3H, CH-phenyl) ppm.

^{13}C NMR (100 MHz, $CDCl_3$): δ = 14.5 (CH_3-9), 18.6 (CH_3-15), 21.0 (CH_3-16), 25.7 (CH_3-13 or 14), 26.7 (CH_3-13 or 14), 48.3 (C-5), 76.6 (CH-2), 77.5 (CH-3), 79.6 (CH-10), 80.3 (CH-4), 114.7 (C-12), 125.9 (CH-phenyl), 128.9 (CH-phenyl), 129.1 (CH-phenyl), 133.5 (C-11), 125.5 (C-7), 173.5 (C-1), 175.8 (C-6) ppm.

IR (thin film): ν 2990, 2929, 1780, 1675, 1457, 1341, 1191, 1069, 1014, 953, 733, 701 cm^{-1}.

HRMS (EI) calcd for $C_{21}H_{25}NO_7$ $[M+Na]^+$, 426.1529; found 426.1527 +/− 5ppm.

Optical Rotation: $[\alpha]^{20}_{D}$ (c 1.0, $CHCl_3$) = −1.9°.

NOE-analysis:

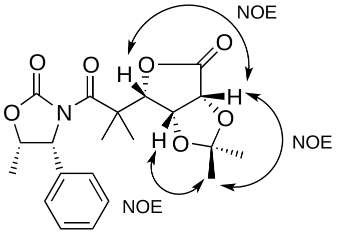

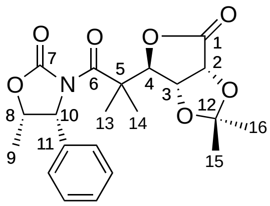

20

(4*R*,5*S*)-3-(2-((3a*R*,4*R*,6a*R*)-2,2-Dimethyl-6-oxotetrahydrofuro[3,4-d][1,3]dioxol-4-yl)-2-methylpropanoyl)-5-methyl-4-phenyloxazolidin-2-one (20). For the preparation of intermediate **20** the same procedure as described above was used starting from diastereomeric alcohol **19** (60 mg, 0.13 mmol). Lactone **20** (14 mg, 27%) was isolated after the two step procedure.

^{1}H NMR (400 MHz, $CDCl_3$): δ = 0.83 (d, *J* = 6.7 Hz 3H, CH_3-9), 1.40 (s, 3H, CH_3-15), 1.49 (s, 3H, CH_3-16), 1.57 (s, 3H, CH_3-13 or 14), 1.67 (s, 3H, CH_3-13 or 14), 4.36 (d, *J* = 1.0 Hz, 1H, H-4), 4.74-4.82 (m, 2H, H-3, H-8), 4.99 (d, *J* = 6.6 Hz, 1H, H-2), 5.67 (d, *J* = 7.6 Hz, 1H, H-10), 7.24-7.45 (m, 5H, CH-phenyl) ppm.

^{13}C NMR (100 MHz, $CDCl_3$): δ = 14.5 (CH_3-9), 19.0 (CH_3-13 or 14), 20.7 (CH_3-13 or 14), 25.4 (CH_3-15), 26.6 (CH_3-16), 47.9 (C-5), 56.6 (CH-3), 76.9 (CH-2), 78.1 (CH-8), 79.3 (CH-10), 92.4 (CH-4), 113.9 (C-12), 125.8 (CH-phenyl), 129.0 (CH-phenyl), 129.1 (CH-phenyl), 133.3 (C-11), 151.6 (C-7), 174.0 (C-1), 175.5 (C-6) ppm.

IR (thin film) ν 2990, 2929, 1780, 1675, 1457, 1341, 1191, 1069, 1014, 953, 733, 701 cm^{-1}.

HRMS (EI) calcd for $C_{21}H_{25}NO_7$ $[M+Na]^+$, 426.1529; found 426.1527 +/− 5ppm.

Optical Rotation: $[\alpha]^{20}_{D}$ (c 0.5, $CHCl_3$) = −3.8°.

NOE-analysis:

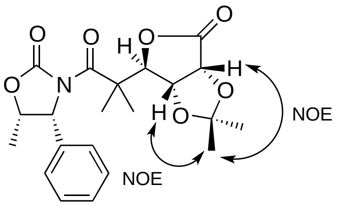

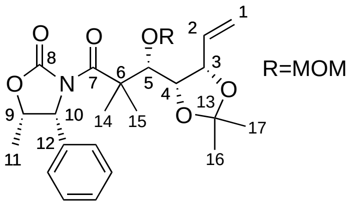

14

(4R,5*S*)-3-((*S*)-3-((4*S*,5*S*)-2,2-Dimethyl-5-vinyl-1,3-dioxolan-4-yl)-3-(methoxymethoxy)-2,2-dimethylpropanoyl)-5-methyl-4-phenyloxazolidin-2-one (14**).** To a cooled solution (0 °C) of alcohol **13** (230 mg, 0.57 mmol, 1.0 eq) in methylene chloride (3.0 mL) was added DIPEA (0.49 mL, 2.85 mmol, 5.0 eq), followed by the slow dropwise addition of MOM-Cl (0.13 mL, 1.71 mmol, 3.0 eq). Next, the cooling bath was removed and the reaction mixture was stirred for twelve hours at room temperature and additional 2.5 hours at 50 °C until no more starting material could be detected by TLC. The reaction mixture was quenched with water and diluted with methylene chloride. The layers were separated and the aqueous phase was extracted three times with methylene chloride. The combined organic extracts were dried over magnesium sulfate, filtered and the solvent was removed under reduced pressure. The resulting crude MOM-protected product was purified by flash column chromatography (hexanes/ethyl acetate 9:1 to 5:1) providing intermediate **14** (237 mg, 93%) as colorless oil.

^{1}H NMR (400 MHz, $CDCl_3$): δ = 0.90 (d, *J* = 6.6 Hz, 3H, CH_3-13), 1.38 (s, 3H, CH_3-14 or 15), 1.44 (s, 3H, CH_3-16 or 17), 1.48 (s, 3H, CH_3-16 or 17), 1.53 (s, 3H, CH_3-14 or 15), 3.36 (s, 3H, OCH_3- MOM), 4.32 (dd, *J* = 3.7, 6.2 Hz, 1H, H-4), 4.57 (dd, *J* = 6.2, 7.5 Hz, 1H, H-3), 4.62 (d, *J* = 6.6 Hz, 1H, CH_2-MOM), 4.68 (d, *J* = 6.6 Hz, 1H, CH_2-MOM), 4.77 (quint, *J* = 6.7 Hz, 1H, H-9), 4.78 (d, *J* = 3.7 Hz, 1H, H-5), 5.28-5.39 (m, 2H, CH_2-1), 5.63 (d, *J* = 7.1 Hz, 1H, H-10), 6.09 (ddd, *J* = 7.5, 10.1, 17.4 Hz, 1H, H-2), 7.27-7.32 (m, 2H, CH-phenyl), 7.34-7.44 (m, 3H, CH-phenyl) ppm.

^{13}C NMR (100 MHz, $CDCl_3$): δ = 14.4 (CH_3-13) 21.3 (CH_3-14 or 15), 21.9 (CH_3-14 or 15), 25.8 (CH_3-16 or 17), 27.5 (CH_3-16 or 17), 50.5 (C-6), 56.7 (OCH_3-MOM), 57.3 (CH-5), 76.6 (CH-9), 78.3 (CH-4), 79.3 (CH-10), 79.9 (CH-3), 98.5 (CH_2-MOM), 108.5 (C-12), 118.8 (CH_2-1), 125.9 (CH-phenyl), 128.8 (CH-phenyl), 128.9 (CH-phenyl), 133.7 (C-11), 134.9 (CH-2), 152.6 (C-8), 176.6 (C-7) ppm.

IR (thin film) ν 2985, 2937, 1776, 1690, 1456, 1369, 1339, 1247, 1191, 1121, 1068, 1032, 882, 769, 700 cm^{-1}.

HRMS (EI) calcd for $C_{24}H_{33}NO_7$ $[M+Na]^+$, 470.2155; found 470.2160 +/− 5ppm.

Optical Rotation: $[\alpha]^{20}_{D}$ (c 1.0, $CHCl_3$) = −11.5°.

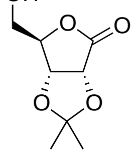

24

(3a*R*,6*R*,6a*R*)-6-(Hydroxymethyl)-2,2-dimethyldihydrofuro[3,4-d][1,3]dioxol-4(3aH)-one (24). D-Ribonolactone (10.0 g, 67.5 mmol) was dissolved in acetone (50 mL) and boron trifluoride etherate (0.855 mL, 6.75 mmol, 0.1 eq) was added to the solution at room temperature, followed by 2,2-dimethoxypropane (10.0 mL). The reaction mixture was stirred for one hour before the solvent was removed under reduced pressure to afford a light brown solid, which was dissolved in ethyl acetate. The resulting solution was extracted with water twice, with brine once, dried over sodium sulfate, filtered and

the solvent was removed under reduced pressure delivering crude lactone **24** (10.93 g, 86%) as light yellow crystals. The product was used without any further purification for the following step.
^{1}H NMR (400 MHz, $CDCl_3$): δ = 1.38 (s, 3H), 1.48 (s, 3H), 2.29 (t, *J* = 5.4 Hz, 1H, OH), 3.81 (ddd, *J* = 1.8, 5.6, 12.1 Hz, 1H), 4.0 (ddd, *J* = 2.5, 5.6, 12.1 Hz, 1H), 4.63 (bt, *J* = 2.0 Hz, 1H), 4.78 (d, *J* = 5.8 Hz, 1H), 4.83 (d, *J* = 5.8 Hz, 1H) ppm.
^{13}C NMR (100 MHz, $CDCl_3$): δ = 25.6 (CH_3), 26.9 (CH_3), 62.2 (CH_2), 75.8 (CH), 78.4 (CH), 82.7 (CH), 113.3 (C), 174.9 (C) ppm.
IR (thin film) ν 3469, 2991, 1767, 1379, 1273, 1222, 1200, 1154, 1093, 975, 856, 810, 774 cm^{-1}.
HRMS (EI) calcd for $C_8H_{12}O_5$ $[M+Na]^+$, 211.0583; found 211.0583 +/− 5ppm.
Optical Rotation: $[\alpha]^{20}_{D}$ (c 1.0, $CHCl_3$) = −66.9°.

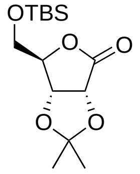

25

(3a*R*,6*R*,6a*R*)-6-(((*tert*-Butyldimethylsilyl)oxy)methyl)-2,2-dimethyldihydrofuro[3,4-d][1,3]dioxol-4(3aH)-one (25**).** Imidazole (2.17 g, 31.9 mmol, 1.2 eq) and TBS-Cl (4.08 g, 27.1 mmol, 1.02 eq) were added to a solution of lactone **24** (5.0 g, 26.6 mmol, 1.0 eq) in DMF (20 mL) at 0 °C. The reaction mixture was allowed to warm to room temperature and was stirred for 16 hours before the reaction was quenched by the addition of water. After separating the two layers the aqueous phase was extracted with diethyl ether three times. The combined organic extracts were dried over magnesium sulfate, filtered and the organic solvents were removed under reduced pressure to afford the crude fully protected ribonolactone (**25**), which was further purified by flash column chromatography (hexanes/ethyl acetate 5:1) to give **25** in 93% yield (7.5 g).
^{1}H NMR (400 MHz, $CDCl_3$): δ = 0.06 (s, 3H), 0.08 (s, 3H), 0.88 (s, 9H), 1.39 (s, 3H), 1.47 (s, 3H), 3.80 (dd, *J* = 1.5, 11.3 Hz, 1H), 3.90 (dd, *J* = 1.5, 11.3 Hz, 1H), 4.58-4.61 (m, 1H), 4.70 (d, *J* = 5.8 Hz, 1H), 4.73 (d, *J* = 5.8 Hz, 1H) ppm.
These spectral characteristics are identical to those previously reported.[3]

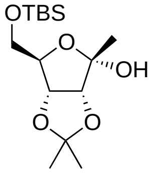

26

(3a*R*,4*S*,6*R*,6a*R*)-6-(((*tert*-Butyldimethylsilyl)oxy)methyl)-2,2,4-trimethyltetrahydrofuro[3,4-d][1,3]dioxol-4-ol (26). A solution of methyllithium (0.68 mL, 1.6 M in Et_2O, 1.1 mmol, 1.1 eq) was added dropwise to a solution of lactone **25** (300 mg, 0.99 mmol, 1.0 eq) in dry THF (3.5 mL). The reaction mixture was stirred for 3.5 hours at −78 °C before it was quenched with water at −78 °C and warmed to room temperature. The product was extracted with ethyl acetate, the combined organic extracts were washed with brine and dried over sodium sulfate. The precipitate was removed by filtration and the solvent was evaporated under reduced pressure. The crude lactole **26** (312 mg, 99%) was used for the following step without any further purification.
^{1}H NMR (400 MHz, $CDCl_3$): δ = 0.14 (s, 3H), 0.15 (s, 3H), 0.93 (s, 9H), 1.34 (s, 3H), 1.50 (s, 3H), 1.52 (d, 3H, *J* = 1.0 Hz), 3.75 (dd, *J* = 2.0, 11.1 Hz, 1H), 3.78 (dd, *J* = 2.0, 11.1 Hz, 1H), 4.25 (dd, *J* = 2.0, 3.5 Hz, 1H), 4.43 (d, *J* = 5.8 Hz, 1H), 4.80 (dd, *J* = 1.5, 5.8 Hz, 1H), 5.11 (bd, *J* = 1.0 Hz, 1H) ppm.
^{13}C NMR (100 MHz, $CDCl_3$): δ = −5.6 (CH_3), −5.5 (CH_3), 18.4 (C), 21.4 (CH_3), 25.3 (CH_3), 25.9 (CH_3), 26.8 (CH_3), 65.1 (CH_2), 82.2 (CH), 86.0 (CH), 88.2 (CH), 106.7 (C), 112.6 (C) ppm.
These spectral characteristics are identical to those reported.[4]

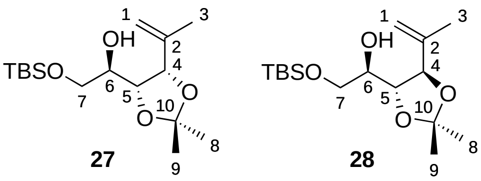

Most conditions applied for the installation of the double bond resulted in α-racemization. Depending on reaction conditions, mixtures containing diastereomeric alkenes **27** and **28** were obtained.
The following procedure allowed the isolation of pure diastereomer **27** in moderate yield.

(*R*)-2-((*tert*-Butyldimethylsilyl)oxy)-1-((4*R*,5*S*)-2,2-dimethyl-5-(prop-1-en-2-yl)-1,3-dioxolan-4-yl)ethanol (27). Methyltriphenylphosphonium bromide (789 mg, 2.2 mmol, 2.2 eq) was dissolved in toluene (6.5 mL) and cooled to 0 °C. *t*-BuOK (248 mg, 2.2 mmol, 2.2 eq) was added in one portion and the resulting yellow reaction mixture was stirred for 30 min at 0 °C and additional three hours at room temperature. The yellow suspension was cooled to −78 °C, lactole **26** (200 mg, 1.0 mmol, 1.0 eq) was added and the reaction mixture was allowed to come to room temperature over twelve hours. The reaction was terminated by the addition of saturated ammonium chloride solution, the layers were separated and the aqueous phase was extracted three times with ethyl acetate. The combined organic extracts were dried over sodium sulfate, filtered and the solvent was removed under reduced pressure. The resulting crude product was purified by flash column chromatography (hexanes/ethyl acetate 19:1) delivering alcohol **27** (58 mg) in 30% yield.

^{1}H NMR (400 MHz, $CDCl_3$): δ = 0.08 (s, 3H, Si-CH_3), 0.081 (s, 3H, Si-CH_3), 0.90 (s, 9H, CH_3-*t*Bu), 1.36 (s, 3H, CH_3-9), 1.47 (s, 3H, CH_3-8), 1.85 (s, 3H, CH_3-3), 2.47 (d, *J* = 4.8 Hz, 1H, OH), 3.56-3.64 (m, 1H, H-6), 3.67 (dd, *J* = 6.3, 9.9 Hz, 1H, H-7), 3.80 (dd, *J* = 3.0, 9.9 Hz, 1H, H-7), 4.07 (dd, *J* = 6.3, 8.8 Hz, 1H, H-5), 6.06 (d, *J* = 6.3 Hz, 1H, H-4), 5.0-5.04 (m, 1H, H-1a), 5.16-5.19 (m, 1H, H-1b) ppm.

^{13}C NMR (100 MHz, $CDCl_3$): δ = −5.2 (Si-CH_3), −5.24 (Si-CH_3), 18.5 (C-tBu), 20.8 (CH_3-3), 25.3 (CH_3-9), 26.1 (CH_3-tBu), 27.4 (CH_3-8), 64.6 (CH_2-7), 69.7 (CH-6), 77.9 (CH-5), 80.4 (CH-4), 108.2 (C-10), 112.5 (CH_2-1), 141.5 (C-2) ppm.

IR (thin film) ν 3565, 2929, 2857, 1463, 1380, 1253, 1165, 1115, 1078, 1057, 899, 833 cm^{-1}.

HRMS (EI) calcd for $C_{16}H_{32}O_4Si$ $[M+Na]^+$, 339.1968; found 339.1970 +/− 5ppm.

Optical Rotation: $[\alpha]^{20}_D$(c 1.0, $CHCl_3$) = +40.8°.

NOE-analysis:

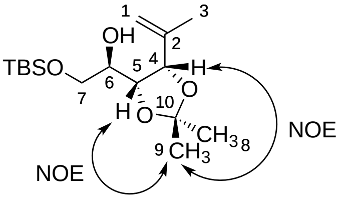

Experimental data for diastereomer 28 (undesired):

^{1}H NMR (400 MHz, $CDCl_3$): δ = 0.08 (s, 3H, Si-CH_3), 0.081 (s, 3H, Si-CH_3), 0.90 (s, 9H, CH_3-*t*Bu), 1.41 (s, 3H, CH_3-8), 1.43 (s, 3H, CH_3-9), 1.81 (s, 3H, CH_3-3), 3.63-3.71 (m, 1H, H-7), 3.71-3.80 (m, 2H, H-7, H-6), 3.83-3.89 (m, 1H, H-5), 4.48 (d, *J* = 7.8 Hz, 1H, H-4), 4.98-5.02 (m, 1H, H-1a), 5.13-5.16 (m, 1H, H-1b) ppm.

^{13}C NMR (100 MHz, $CDCl_3$): δ = −5.29 (Si-CH_3), −5.23 (Si-CH_3), 17.7 (CH_3-3), 18.4 (C-*t*Bu), 26.0 (CH_3-tBu), 27.2 (CH_3-9), 27.3 (CH_3-8), 64.1 (CH_2-7), 73.0 (CH-5), 78.3 (CH-6), 82.9 (CH-4), 109.2 (C-10), 115.1 (CH_2-1), 142.6 (C-2) ppm.

IR (thin film) ν 3487, 2930, 2859, 1463, 1370, 1253, 1167, 1060, 902, 836, 778 cm^{-1}.

HRMS (EI) calcd for $C_{16}H_{32}O_4Si$ $[M+Na]^+$, 339.1968; found 339.1967 +/− 5ppm.

Optical Rotation: $[\alpha]^{20}_D$(c 0.5, $CHCl_3$) = −3.4°.

NOE-analysis:

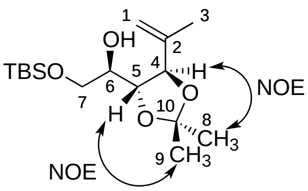

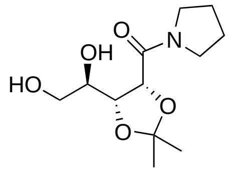

29

((4*R*,5*R*)-5-((*R*)-1,2-Dihydroxyethyl)-2,2-dimethyl-1,3-dioxolan-4-yl)(pyrrolidin-1-yl)methanone (29). Protected ribonolactone **24** (500 mg, 2.7 mmol, 1.0 eq) was dissolved in toluene (11 mL). After the addition of pyrrolidine (1.1 mL, 13.5 mmol, 5.0 eq) the reaction mixture was heated to reflux for twelve hours. The reaction mixture was then cooled to room temperature and the solvent was removed under reduced pressure. The crude material was purified by flash column chromatography (methylene chloride/methanol 19:1) to give amide **29** (610 mg) in 87% yield.

^{1}H NMR (400 MHz, $CDCl_3$): δ = 1.37 (s, 3H), 1.52 (s, 3H), 1.79-2.03 (m, 4H), 2.35 (bs, 1H, OH), 3.43-3.59 (m, 2H), 3.60-3.72 (m, 3H), 3.77-3.89 (m, 2H), 4.27 (dd, *J* = 6.3, 8.8 Hz, 1H), 4.59 (bs, 1H, OH), 4.84 (d, *J* = 6.3 Hz, 1H) ppm.

^{13}C NMR (100 MHz, $CDCl_3$): δ = 23.8 (CH_2), 25.3 (CH_3), 26.5 (CH_2), 27.2 (CH_3), 47.02 (CH_2), 47.14 (CH_2), 64.5 (CH_2), 70.0 (CH), 76.7 (CH), 78.3 (CH), 109.9 (C), 167.8 (C) ppm.

IR (thin film) ν 3335, 2981, 2876, 1781, 1630, 1454, 1372, 1214, 1164, 1053, 907, 726 cm^{-1}.

HRMS (EI) calcd for $C_{12}H_{21}O_5NH$ $[M+H]^+$, 260.1498; found 260.1492 +/− 5ppm.

Optical Rotation: $[\alpha]^{20}_{D}$(c 1.0, $CHCl_3$) = +16.2°.

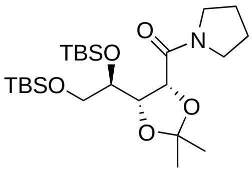

30

((4*R*,5*S*)-2,2-Dimethyl-5-((*R*)-2,2,3,3,8,8,9,9-octamethyl-4,7-dioxa-3,8-disiladecan-5-yl)-1,3-dioxolan-4-yl)(pyrrolidin-1-yl)methanone (30). To a solution of diol **29** (250 mg, 0.96 mmol, 1.0 eq) in methylene chloride (2 mL) were sequentially added 2,6-lutidine (0.335 mL, 2.88 mmol, 3.0 eq) and TBS-triflate (0.706 mL, 3.07 mmol, 3.2 eq) at 0 °C. The cooling bath was removed after the addition and the reaction mixture was allowed to stir at room temperature for 15 hours. The reaction was quenched by the addition of saturated sodium bicarbonate solution. The layers were separated and the aqueous phase was extracted three times with ethyl acetate. The combined organic extracts were dried over solid sodium sulfate, filtered and the solvent was removed under reduced pressure. The crude product was purified by flash column chromatography (hexanes/ethyl acetate 9:1) delivering **30** (375 mg) in 80% yield.

^{1}H NMR (400 MHz, $CDCl_3$): δ = 0.01 (s, 3H), 0.04 (s, 3H), 0.05 (s, 3H), 0.07 (s, 3H), 0.85 (s, 9H), 0.90 (s, 9H), 1.35 (s, 3H), 1.48 (s, 3H), 1.74-2.0 (m, 4H), 3.36-3.55 (m, 3H), 3.60-3.69 (m, 1H), 3.74-3.77 (m, 2H), 4.37-4.41 (m, 2H), 4.67-4.70 (m, 1H) ppm.

^{13}C NMR (100 MHz, $CDCl_3$): δ = −5.4 (CH_3), −5.2 (CH_3), −4.5 (CH_3), −3.7 (CH_3), 18.3 (C), 18.6 (C), 24.2 (CH_2), 26.0 (CH_3), 26.05 (CH_3), 26.1 (CH_3), 26.4 (CH_2), 27.0 (CH_3), 46.1 (CH_2), 46.6 (CH_2), 64.7 (CH_2), 72.4 (CH), 74.6 (CH), 77.6 (CH), 110.4 (C), 167.3 (C) ppm.

IR (thin film) ν 2953, 2929, 2856, 1655, 1442, 1368, 1342, 1250, 1223, 1090, 992, 938, 831, 774, 675 cm^{-1}.

HRMS (EI) calcd for $C_{24}H_{49}NO_5Si_2$ $[M+Na]^+$, 510.3047; found 510.3048 +/− 5ppm.

Optical Rotation: $[\alpha]^{20}_{D}$(c 1.0, $CHCl_3$) = −46.7°.

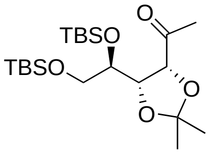

S3

1-((4*R*,5*S*)-2,2-Dimethyl-5-((*R*)-2,2,3,3,8,8,9,9-octamethyl-4,7-dioxa-3,8-disiladecan-5-yl)-1,3-dioxolan-4-yl)ethanone (S3). A solution of methyllithium (1.6 M, 12.8 mL, 20.4 mmol, 2.0 eq) was added to amide **30** (5 g, 10.2 mmol, 1.0 eq) in THF (50 mL) at −78 °C. The reaction mixture was stirred for 15 min when TLC-control showed total consumption of the starting material. The reaction was then terminated by the addition of water. The layers were separated and the aqueous phase was extracted three times with ethyl acetate. The combined organic extracts were dried over sodium sulfate and the solvent was removed under reduced pressure. Further purification of crude **S3** by flash column chromatography (hexanes/ethyl acetate 40:1) delivered ketone **S3** (4.23 g) in 95% yield.

^{1}H NMR (400 MHz, $CDCl_3$): δ = 0.06 (s, 3H), 0.07 (s, 3H), 0.075 (s, 3H), 0.08 (s, 3H), 0.87 (s, 9H), 0.89 (s, 9H), 1.36 (s, 3H), 1.60 (s, 3H), 2.31 (s, 3H), 3.57 (dd, *J* = 5.1, 10.4 Hz, 1H), 3.74 (dd, *J* = 7.3, 10.4 Hz, 1H), 4.05 (ddd, *J* = 3.4, 5.1, 7.3 Hz, 1H), 4.38 (d, *J* = 7.8 Hz, 1H), 4.57 (dd, *J* = 3.4, 7.8 Hz, 1H) ppm.

^{13}C NMR (100 MHz, $CDCl_3$): δ = −5.3 (CH_3), −4.8 (CH_3), −4.1 (CH_3), 18.4 (C), 18.6 (C), 24.7 (CH_3), 26.1 (CH_3), 26.15 (CH_3), 26.7 (CH_3), 29.2 (CH_3), 63.9 (CH_2), 72.6 (CH), 79.8 (CH), 80.6 (CH), 109.1 (C), 209.3 (C) ppm.

IR (thin film) ν 2930, 2887, 2858, 1717, 1473, 1361, 1253, 1214, 1150, 1082, 939, 832, 776, 669 cm^{-1}.

HRMS (EI) calcd for $C_{21}H_{44}O_5Si_2$ $[M+Na]^+$, 455.2625; found 455.2620 +/− 5ppm.

Optical Rotation: $[\alpha]^{20}_{D}$(c 1.0, $CHCl_3$) = −20.2°.

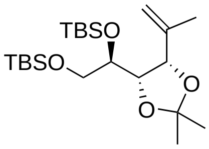

31

(*R*)-5-((4*S*,5*S*)-2,2-Dimethyl-5-(prop-1-en-2-yl)-1,3-dioxolan-4-yl)-2,2,3,3,8,8,9,9-octamethyl-4,7-dioxa-3,8-disiladecane (31). Ketone **S3** (2.8 g, 6.5 mmol, 1.0 eq) was dissolved in freshly distilled THF (40 mL) and cooled to 0 °C. A solution of Tebbe-reagent (15.6 mL, 0.5 M in toluene, 7.8 mmol, 1.2 eq) was slowly added *via* syringe. The reaction mixture was stirred for one hour at 0 °C before it was quenched by the addition of a saturated sodium bicarbonate solution. After the separation of the two layers, the aqueous phase was extracted with ethyl acetate three times. The combined organic extracts were dried over solid sodium sulfate, filtered and the solvent was removed under reduced pressure. Crude alkene **31** was further purified by flash column chromatography (hexanes/ethyl acetate 40:1) delivering 2.32 g (83%) of the desired intermediate as light yellow oil.

^{1}H NMR (400 MHz, $CDCl_3$): δ = 0.04 (s, 6H), 0.041 (s, 3H), 0.08 (s, 3H), 0.88 (s, 9H), 0.89 (s, 9H), 1.35 (s, 3H), 1.48 (s, 3H), 1.78 (s, 3H), 3.64-3.75 (m, 3H), 4.28 (dd, *J* = 4.8, 7.1 Hz, 1H), 4.61 (d, *J* = 7.1 Hz, 1H), 4.95-4.98 (m, 1H), 5.07-5.10 (m, 1H) ppm.

^{13}C NMR (100 MHz, $CDCl_3$): δ = −5.3 (CH_3), −4.3 (CH_3), −3.7 (CH_3), 18.50 (C), 18.59 (C), 20.4 (CH_3), 25.1 (CH_3), 26.1 (CH_3), 26.2 (CH_3), 26.6 (CH_3), 65.4 (CH_2), 73.2 (CH), 79.4 (CH), 80.5 (CH), 107.9 (C), 113.9 (CH_2), 141.0 (C) ppm.

IR (thin film) ν 2954, 2857, 1492, 1463, 1368, 1252, 1211, 1143, 1087, 1040, 1002, 986, 965, 947, 830, 773, 665 cm^{-1}.

HRMS (EI) calcd for $C_{22}H_{46}O_4Si_2$ $[M+Na]^+$, 453.2833; found 453.2834 +/− 5ppm.

Optical Rotation: $[\alpha]^{20}_{D}$(c 1.0, $CHCl_3$) = +30.1°.

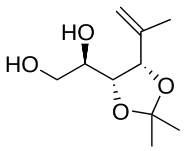

S4

(*R*)-1-((4*R*,5*S*)-2,2-Dimethyl-5-(prop-1-en-2-yl)-1,3-dioxolan-4-yl)ethane-1,2-diol (S4). TBAF (1.59 mL, 1 M in THF, 1.59 mmol, 3.0 eq) was added to a solution of TBS-protected diol **31** (230 mg, 0.53 mmol, 1.0 eq) in THF (3 mL) at 0 °C. After the addition the cooling bath was removed and the reaction mixture was stirred for 3.5 hours at room temperature. The reaction was then quenched by the addition of saturated ammonium chloride solution. The two layers were separated and the aqueous layer was extracted with ethyl acetate three times. The organic extracts were dried over sodium sulfate, filtered and the solvent was removed under reduced pressure. The crude diol was purified by flash column chromatography (hexanes/ethyl acetate 5:1 to 1:1) providing **S4** (107 mg) in quantitative yield.

^{1}H NMR (400 MHz, $CDCl_3$): δ = 1.37 (s, 3H), 1.49 (s, 3H), 1.87 (s, 3H), 2.06 (dd, *J* = 5.3, 6.8 Hz, OH), 2.14 (d, *J* = 4.8 Hz, OH), 3.65-3.74 (m, 2H), 3.76-3.86 (m, 1H), 4.08-4.16 (m, 1H), 4.66 (d, *J* = 6.1 Hz, 1H), 5.06-5.09 (m, 1H), 5.24-5.27 (m, 1H) ppm.

^{13}C NMR (100 MHz, $CDCl_3$): δ = 20.9 (CH_3), 25.4 (CH_3), 27.5 (CH_3), 64.5 (CH_2), 69.9 (CH), 78.2 (CH), 80.0 (CH), 108.4 (C), 112.9 (CH_2), 141.7 (C) ppm.

IR (thin film) ν 3369, 2986, 2935, 1652, 1380, 1234, 1212, 1164, 1038, 900, 875, 799 cm^{-1}.

HRMS (EI) calcd for $C_{10}H_{18}O_4$ $[M+Na]^+$, 225.1103; found 225.1100 +/− 5ppm.

Optical Rotation: $[\alpha]^{20}_{D}$(c 1.0, $CHCl_3$) = +80.4°.

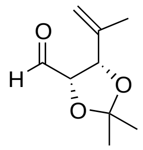

32

(4*S*,5*S*)-2,2-Dimethyl-5-(prop-1-en-2-yl)-1,3-dioxolane-4-carbaldehyde (32). To a solution of diol **S4** (900 mg, 4.44 mmol, 1.0 eq) in methylene chloride (24 mL) was added a solution of sodium periodate (1.42 g, 6.66 mmol, 1.5 eq) in water (12 mL) at 0 °C. The reaction was stirred for two hours at 0 °C before it was diluted with methylene chloride and water. The layers were separated and the aqueous phase was extracted with methylene chloride three times. The combined organic extracts were dried over sodium sulfate, filtered and the solvent was removed *in vacuo*. The crude aldehyde was further purified by flash column chromatography (pentanes/diethyl ether 5:1) giving **32** (683 mg) in 90% yield.

^{1}H NMR (400 MHz, $CDCl_3$): δ = 1.44 (s, 3H), 1.64 (s, 3H), 1.69 (s, 3H), 4.37 (dd, *J* = 3.5, 7.6 Hz, 1H), 4.79 (d, *J* = 7.6 Hz, 1H), 4.98-5.01 (m, 1H), 5.19-5.22 (m, 1H), 9.44 (d, *J* = 3.5 Hz, 1H) ppm.

^{13}C NMR (100 MHz, $CDCl_3$): δ = 19.7 (CH_3), 25.3 (CH_3), 27.2 (CH_3), 80.9 (CH), 81.6 (CH), 111.3 (C), 113.7 (CH_2), 138.1 (C), 199.9 (CHO) ppm.

IR (thin film) ν 2989, 2939, 1732, 1655, 1450, 1381, 1255, 1215, 1159, 1078, 907, 858, 795, 744 cm^{-1}.

HRMS (EI) calcd for $C_9H_{14}O_3$ $[M+Na]^+$, 193.0841; found 193.0848 +/− 5ppm.

Optical Rotation: $[\alpha]^{20}_{D}$(c 1.0, $CHCl_3$) = +55.0°.

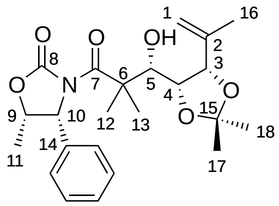

33

(4*R*,5*S*)-3-((*S*)-3-((4*R*,5*S*)-2,2-Dimethyl-5-(prop-1-en-2-yl)-1,3-dioxolan-4-yl)-3-hydroxy-2,2-dimethylpropanoyl)-5-methyl-4-phenyloxazolidin-2-one (33). A solution of SmI_2 (100 mL, 0.1 M in THF, 10 mmol, 2.5 eq) was cannulated into a 250 mL round bottom Schlenk flask which was precooled to −78 °C. A solution of bromide **7** (1.44 g, 4.41 mmol, 1.1 eq) and aldehyde **32** (683 mg, 4.01 mmol, 1.0 eq) in 60 mL degassed THF (3 pump freeze thaw cycles) was added to the SmI_2 solution *via* cannula.

The reaction mixture was stirred for one hour at −78 °C before it was quenched by the addition of aqueous saturated solutions of sodium thiosulfate (50 mL) and sodium bicarbonate (50 mL) at −78 °C. The biphasic system was allowed to warm to room temperature. The two phases were separated, and the aqueous layer was extracted with ethyl acetate three times. The combined organic extracts were dried over sodium sulfate, filtered and the organic solvents were removed under reduced pressure delivering alcohol **33** as light yellow oil which was further purified by flash column chromatography (hexanes/ethyl acetate 9:1) providing **33** (1.14 g) in 68% yield.

^{1}H NMR (400 MHz, $CDCl_3$): δ = 0.91 (d, *J* = 6.6 Hz, 3H, CH_3-11), 1.36 (s, 3H, CH_3-12 or 13), 1.39 (s, 3H, CH_3-17), 1.43 (s, 3H, CH_3-12 or 13), 1.56 (s, 3H, CH_3-18), 1.87 (s, 3H, CH_3-16), 2.93 (d, *J* = 8.6 Hz, 1H, OH), 4.29 (d, *J* = 7.6 Hz, 1H, H-4), 4.42 (d, *J* = 8.6 Hz, 1H, H-5), 4.71 (d, *J* = 7.6 Hz, 1H, H-3), 4.79 (quint, *J* = 6.6 Hz, 1H, H-9), 5.08-5.12 (m, 1H, H-1a), 5.16-5.20 (m, 1H, H-1b), 5.64 (d, *J* = 7.1 Hz, 1H, H-10), 7.28-7.33 (m, 2H, phenyl), 7.33-7.46 (m, 3H, phenyl) ppm.

^{13}C NMR (100 MHz, $CDCl_3$): δ = 14.2 (CH_3-11), 19.9 (CH_3-12 or 13), 20.0 (CH_3-16), 22.4 (CH_3-12 or 13), 25.0 (CH_3-17), 26.4 (CH_3-18), 50.8 (C-6), 57.6 (CH-9), 70.5 (CH-5), 76.0 (CH-4), 79.3 (CH-10), 81.2 (CH-3), 108.8 (C-15), 112.9 (CH_2-1), 125.9 (CH-phenyl), 128.8 (CH-phenyl), 128.9 (CH-phenyl), 133.7 (C-14), 141.3 (C-2), 152.6 (C-8), 176.7 (C-7) ppm.

IR (thin film) n 3330, 2987, 2937, 1777, 1689, 1456, 1340, 1254, 1214, 1191, 1152, 1120, 972, 950, 905, 768, 700 cm^{-1}.

HRMS (EI) calcd for $C_{23}H_{31}O_6N$ $[M+Na]^+$, 440.2049; found 440.2039 +/− 5ppm.

Optical Rotation: $[\alpha]^{20}_{D}$(c 1.0, $CHCl_3$) = +55.6°.

NOE-analysis:

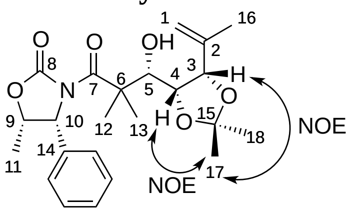

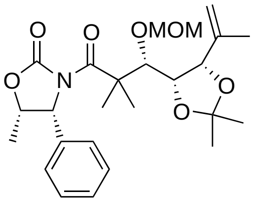

34

(4*R*,5*S*)-3-((*S*)-3-((4*R*,5*S*)-2,2-Dimethyl-5-(prop-1-en-2-yl)-1,3-dioxolan-4-yl)-3-(methoxymethoxy)-2,2-dimethylpropanoyl)-5-methyl-4-phenyloxazolidin-2-one (34). Alcohol **33** (1.0 g, 2.4 mmol, 1.0 eq) was dissolved in methylene chloride (8 mL) and cooled to 0 °C. DIPEA (2.1 mL, 12.0 mmol, 5.0 eq) and MOM-Cl (0.54 mL, 7.2 mmol, 3.0 eq) were added sequentially. After the addition, the ice bath was removed, the reaction mixture was heated to 50 °C and the yellow solution was stirred for 48 hours. The reaction was quenched by the addition of water. After separating the two layers the aqueous phase was extracted with methylene chloride three times. The combined organic extracts were dried over magnesium sulfate, filtered and the solvent was removed under reduced pressure. After purification by flash column chromatography (hexanes/ethyl acetate 9:1 to 5:1) 879 mg (79%) of the MOM protected alkene (**34**) could be isolated as colorless oil.

^{1}H NMR (400 MHz, $CDCl_3$): δ = 0.91 (d, *J* = 6.8 Hz, 3H), 1.40 (s, 3H), 1.46 (s, 3H), 1.48 (s, 3H), 1.58 (s, 3H), 1.86 (s, 3H), 3.26 (s, 3H), 4.46 (dd, *J* = 1.5, 6.8 Hz, 1H), 4.48 (d, *J* = 6.4 Hz, 1H), 4.57 (d, *J* = 6.4 Hz, 1H), 4.62 (d, *J* = 6.8 Hz, 1H), 4.68 (d, *J* = 1.5 Hz, 1H), 4.77 (quint, *J* = 6.8 Hz, 1H), 5.0-5.02 (m, 1H), 5.15-5.17 (m, 1H), 5.65 (d, *J* = 7.2 Hz, 1H), 7.28-7.33 (m, 2H), 7.34-7.44 (m, 3H) ppm.

^{13}C NMR (100 MHz, $CDCl_3$): δ = 14.3 (CH_3), 20.4 (CH_3), 21.9 (CH_3), 22.2 (CH_3), 26.0 (CH_3), 26.3 (CH_3), 50.8 (C), 56.3 (OCH_3), 57.7 (CH), 77.5 (CH), 77.9 (CH), 79.4 (CH), 81.0 (CH), 99.3 (CH_2), 108.2 (C), 112.6 (CH_2), 125.8 (CH), 128.8 (CH), 128.82 (CH), 133.7 (C), 140.6 (C), 152.6 (C), 176.6 (C) ppm.

IR (thin film) ν 2985, 2939, 1776, 1693, 1455, 1367, 1340, 1247, 1192, 1158, 1121, 1083, 1033, 948, 904 cm^{-1}.
HRMS (EI) calcd for $C_{25}H_{35}O_7N$ $[M+Na]^+$, 484.2311; found 484.2309 +/− 5ppm.
Optical Rotation: $[\alpha]^{20}_D$(c 1.0, $CHCl_3$) = −0.9°.

References

[1] a) H. R. Moon, W. J. Choi, H. O. Kim, L. S. Jeong, *Tetrahedron: Asymmetry* **2002**, *13*, 1189-1193; b) S. Pedatella, A. Guaragna, D. D'Alonzo, M. De Nisco, G. Palumbo, *Synthesis* **2006**, 305-308.

[2] W. J. Choi, H. R. Moon, H. O. Kim, B. N. Yoo, J. A. Lee, D. H. Shin, L. S. Jeong, *J. Org. Chem.* **2004**, *69*, 2634-2636.

[3] N. A. Jones, S. F. Jenkinson, R. Soengas, M. Fanefjord, M. R. Wormald, R. A. Dwek, G. P. Kiran, R. Devendar, G. Takata, K. Morimoto, K. Izumori, G. W. J. Fleet, *Tetrahedron: Asymmetry* **2007**, *18*, 774-786.

[4] P. Ciuffreda, B. Buzzi, L. Alessandrini, E. Santaniello, *Eur. J. Org. Chem.* **2004**, 4405-4409.

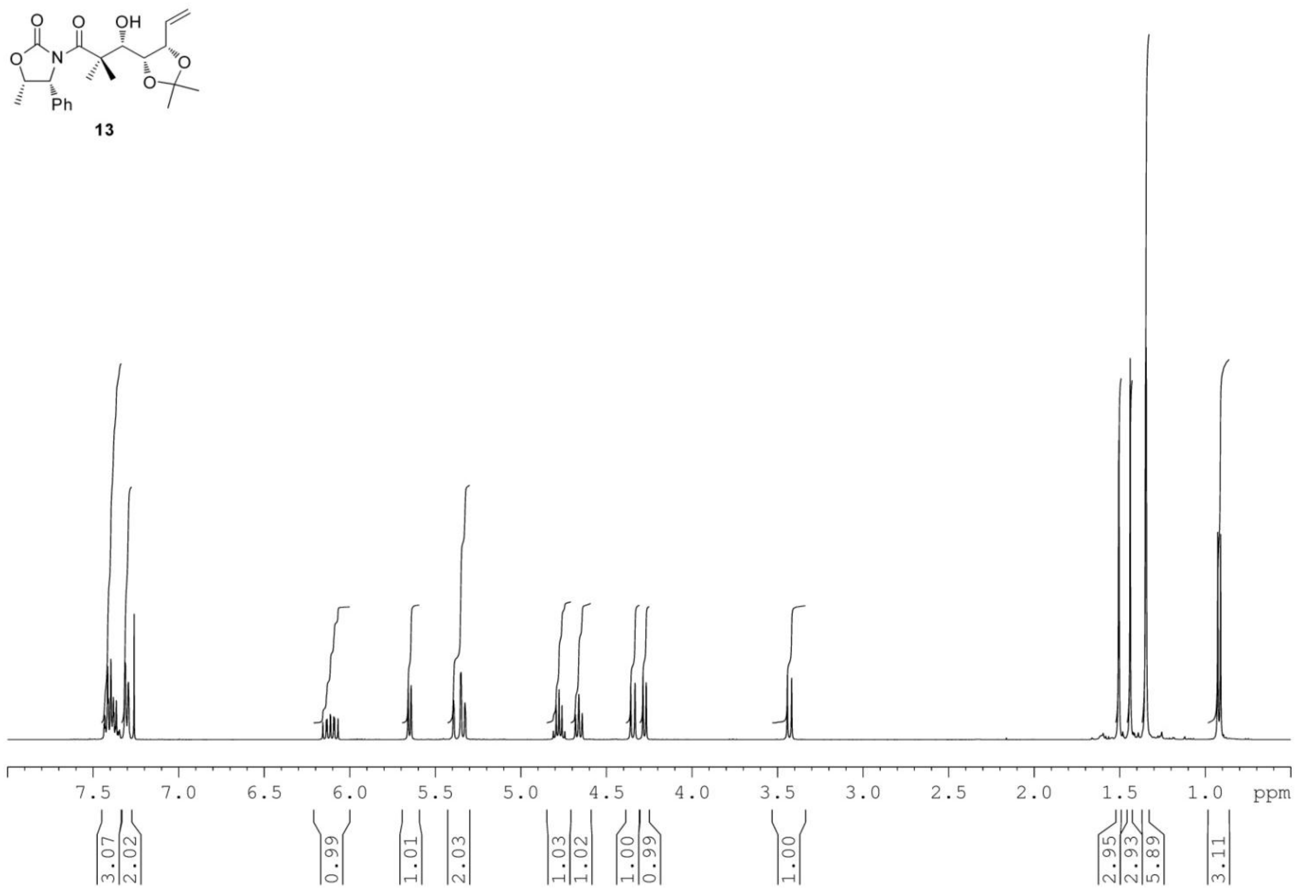
OH
O
N
Ph
13
7.5 7.0 6.5 6.0 5.5 5.0 4.5 4.0 3.5 3.0 2.5 2.0 1.5 1.0 ppm
3.07 2.02 0.99 1.01 2.03 1.03 1.02 1.00 0.99 1.00 2.95 2.93 5.89 3.11

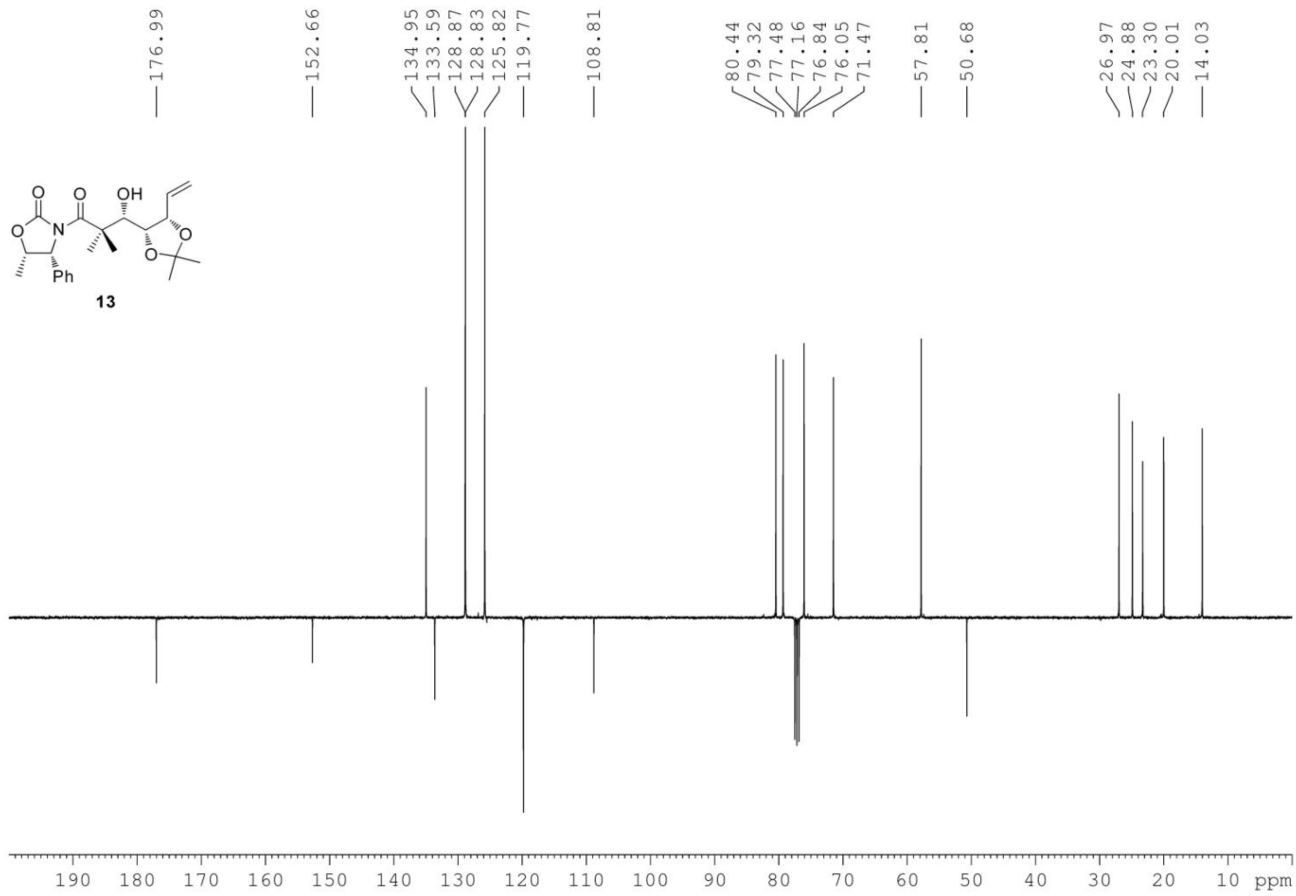
176.99
152.66
134.95 133.59 128.87 128.83 125.82 119.77
108.81
80.44 79.32 77.48 77.16 76.84 76.05 71.47
57.81
50.68
26.97 24.88 23.30 20.01 14.03
OH
O
N
Ph
13
190 180 170 160 150 140 130 120 110 100 90 80 70 60 50 40 30 20 10 ppm

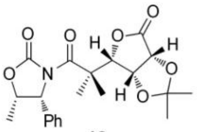

18

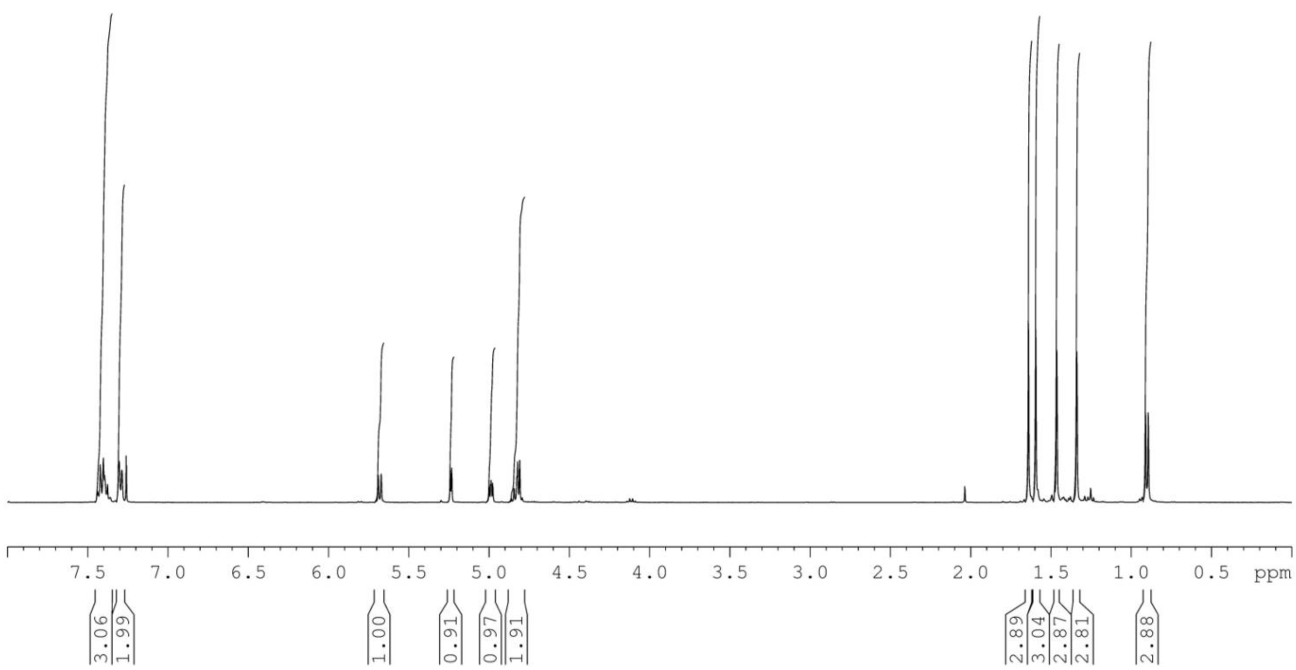

7.5 7.0 6.5 6.0 5.5 5.0 4.5 4.0 3.5 3.0 2.5 2.0 1.5 1.0 0.5 ppm
3.06 1.99 1.00 0.91 0.97 1.91 2.89 3.04 2.87 2.81 2.88

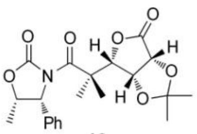

18

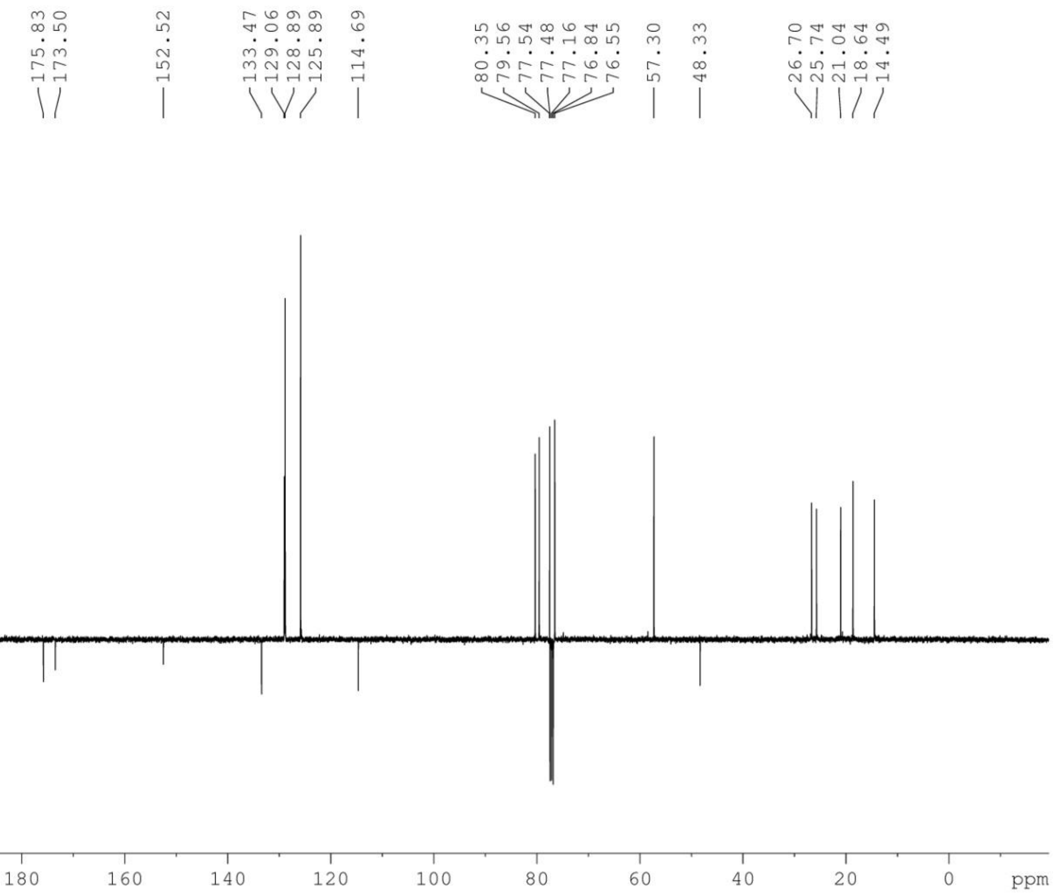

175.83 173.50 152.52 133.47 129.06 128.89 125.89 114.69 80.35 79.56 77.54 77.48 77.16 76.84 76.55 57.30 48.33 26.70 25.74 21.04 18.64 14.49
220 200 180 160 140 120 100 80 60 40 20 0 ppm

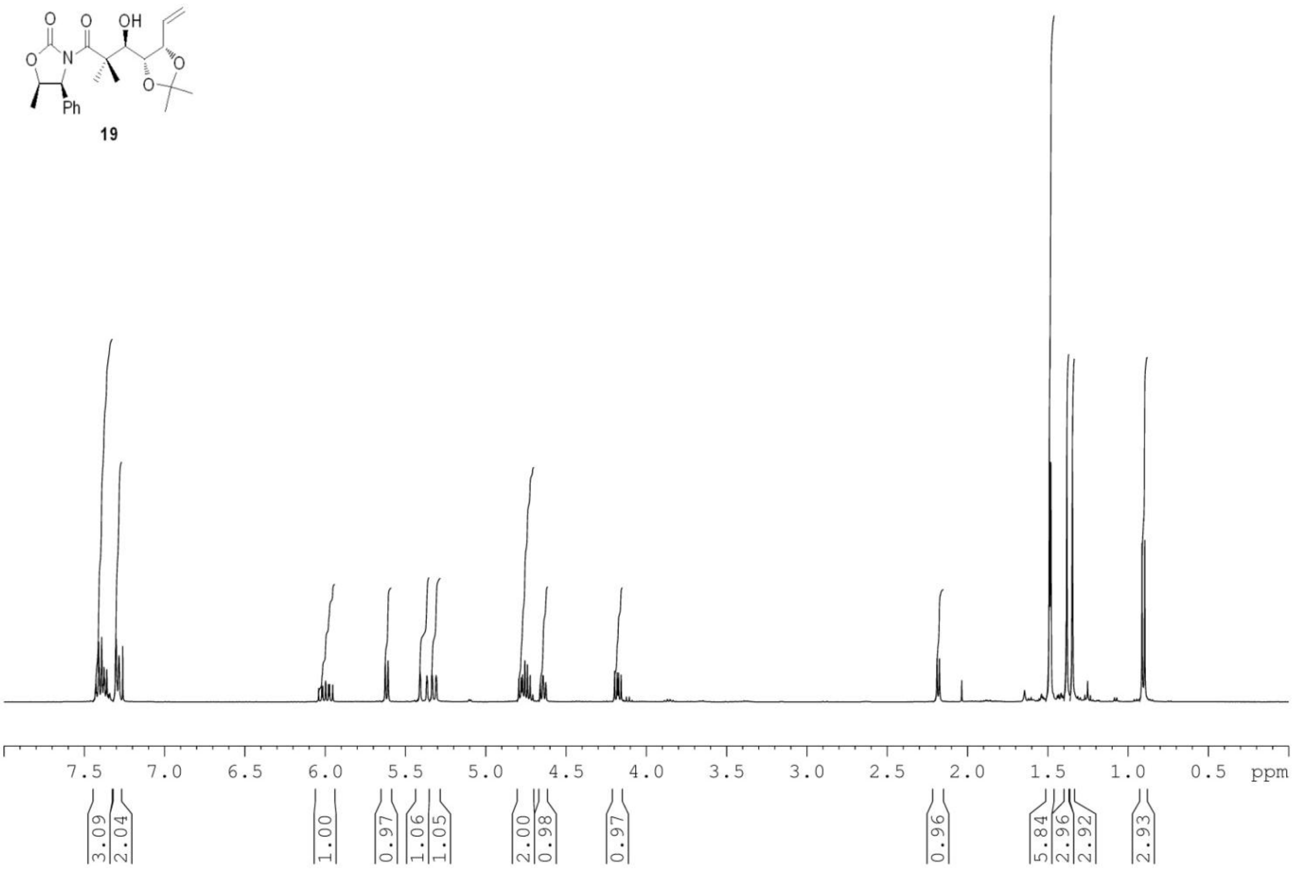
19
7.5 7.0 6.5 6.0 5.5 5.0 4.5 4.0 3.5 3.0 2.5 2.0 1.5 1.0 0.5 ppm
3.09 2.04 1.00 0.97 1.06 1.05 2.00 0.98 0.97 0.96 5.84 2.96 2.92 2.93

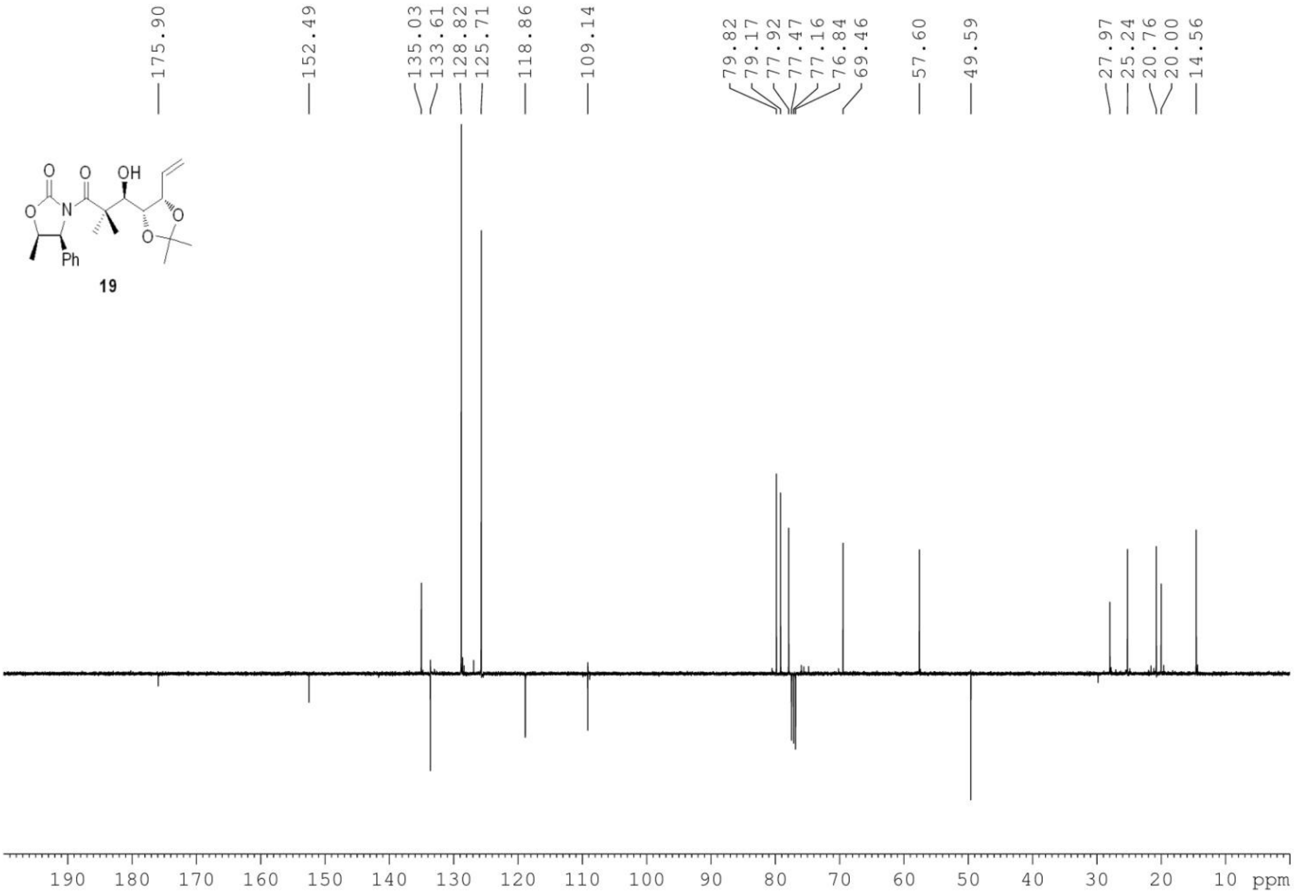
175.90
152.49
135.03 133.61 128.82 125.71
118.86
109.14
79.82 79.17 77.92 77.47 77.16 76.84 69.46
57.60
49.59
27.97 25.24 20.76 20.00 14.56
19
190 180 170 160 150 140 130 120 110 100 90 80 70 60 50 40 30 20 10 ppm

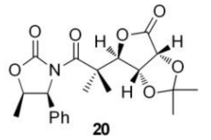

20

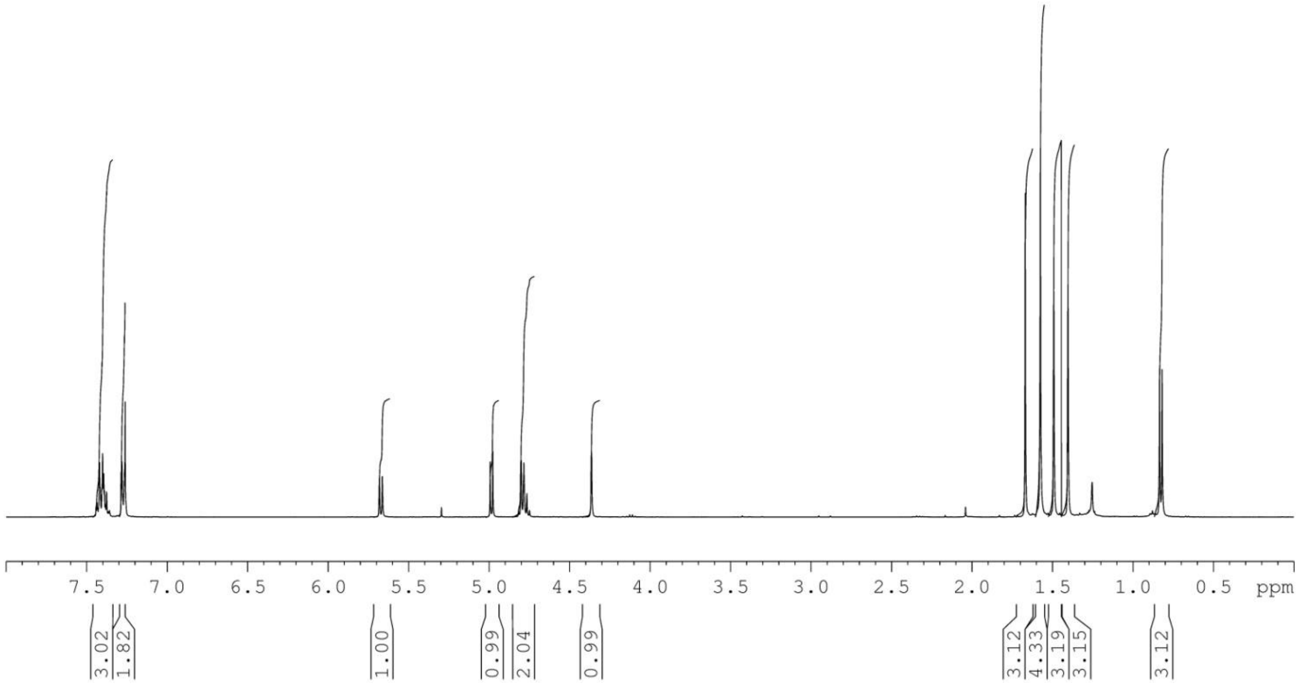
7.5 7.0 6.5 6.0 5.5 5.0 4.5 4.0 3.5 3.0 2.5 2.0 1.5 1.0 0.5 ppm
3.02
1.82
1.00
0.99
2.04
0.99
3.12
4.33
3.19
3.15
3.12

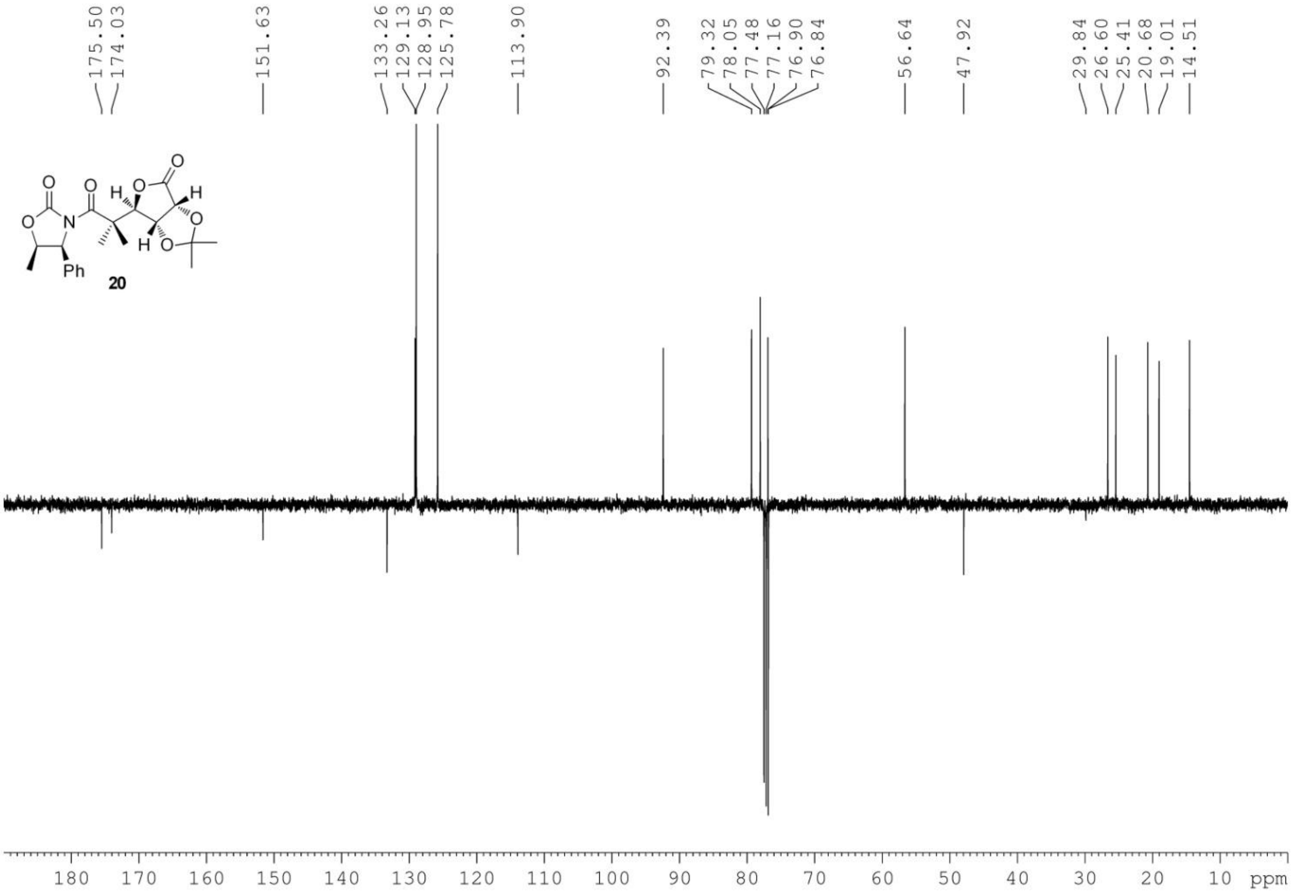
175.50
174.03
151.63
133.26
129.13
128.95
125.78
113.90
92.39
79.32
78.05
77.48
77.16
76.90
76.84
56.64
47.92
29.84
26.60
25.41
20.68
19.01
14.51
Ph
20
180 170 160 150 140 130 120 110 100 90 80 70 60 50 40 30 20 10 ppm

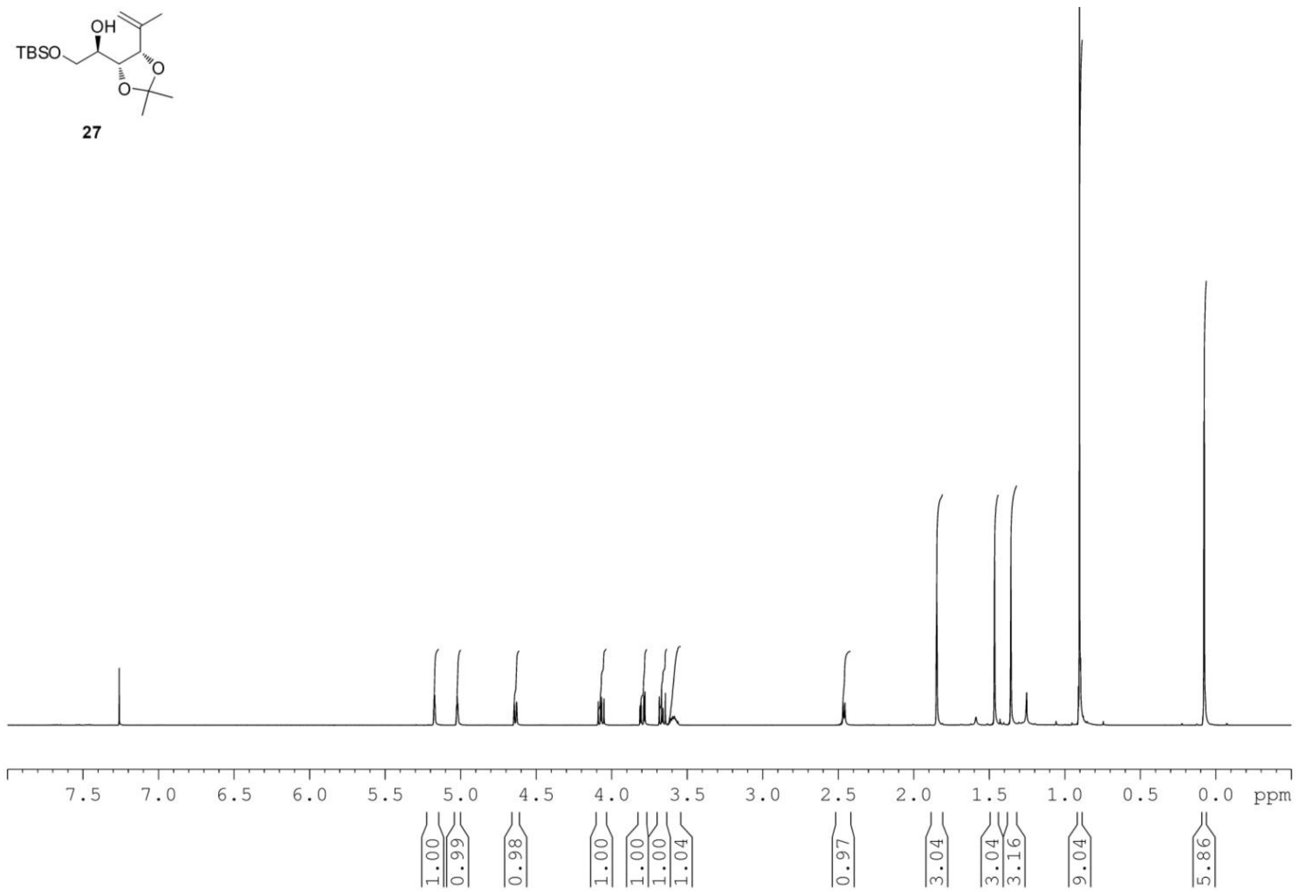
TBSO
OH
O
O
27
7.5 7.0 6.5 6.0 5.5 5.0 4.5 4.0 3.5 3.0 2.5 2.0 1.5 1.0 0.5 0.0 ppm
1.00 0.99 0.98 1.00 1.00 1.00 1.04 0.97 3.04 3.04 3.16 9.04 5.86

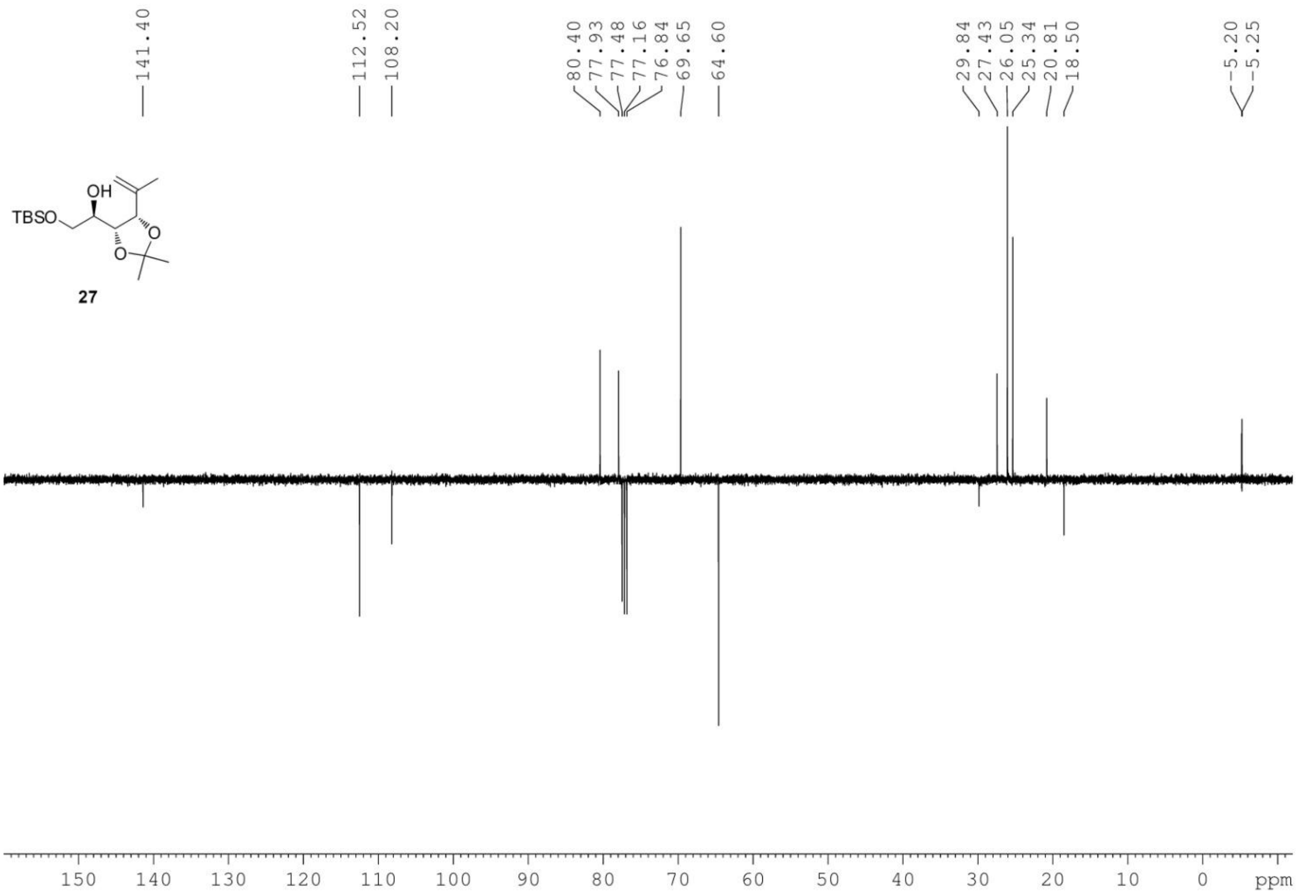
141.40
112.52
108.20
80.40
77.93
77.48
77.16
76.84
69.65
64.60
29.84
27.43
26.05
25.34
20.81
18.50
-5.20
-5.25
TBSO
OH
O
O
27
150 140 130 120 110 100 90 80 70 60 50 40 30 20 10 0 ppm

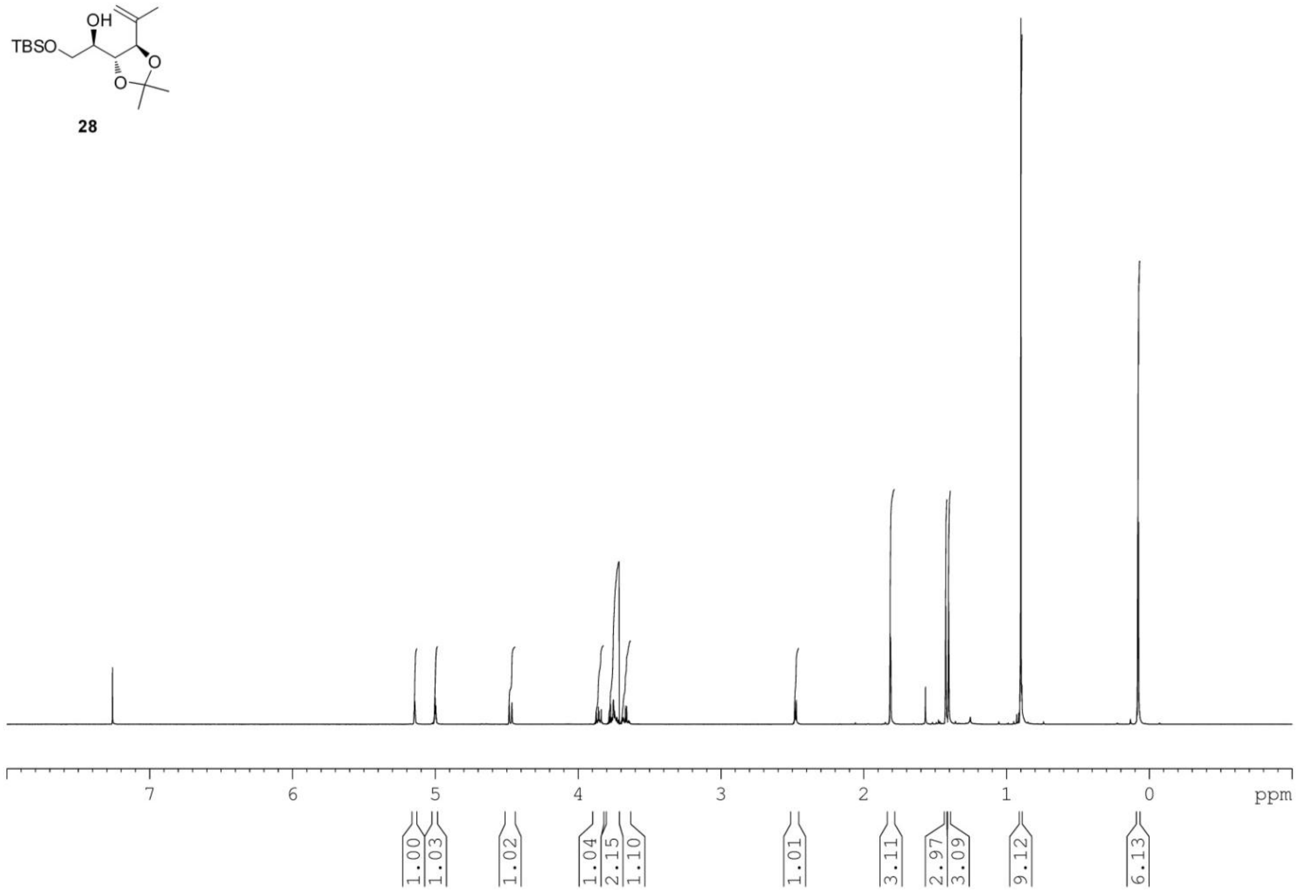
TBSO
OH
O
O
28
7
6
5
4
3
2
1
0
ppm
1.00
1.03
1.02
1.04
2.15
1.10
1.01
3.11
2.97
3.09
9.12
6.13

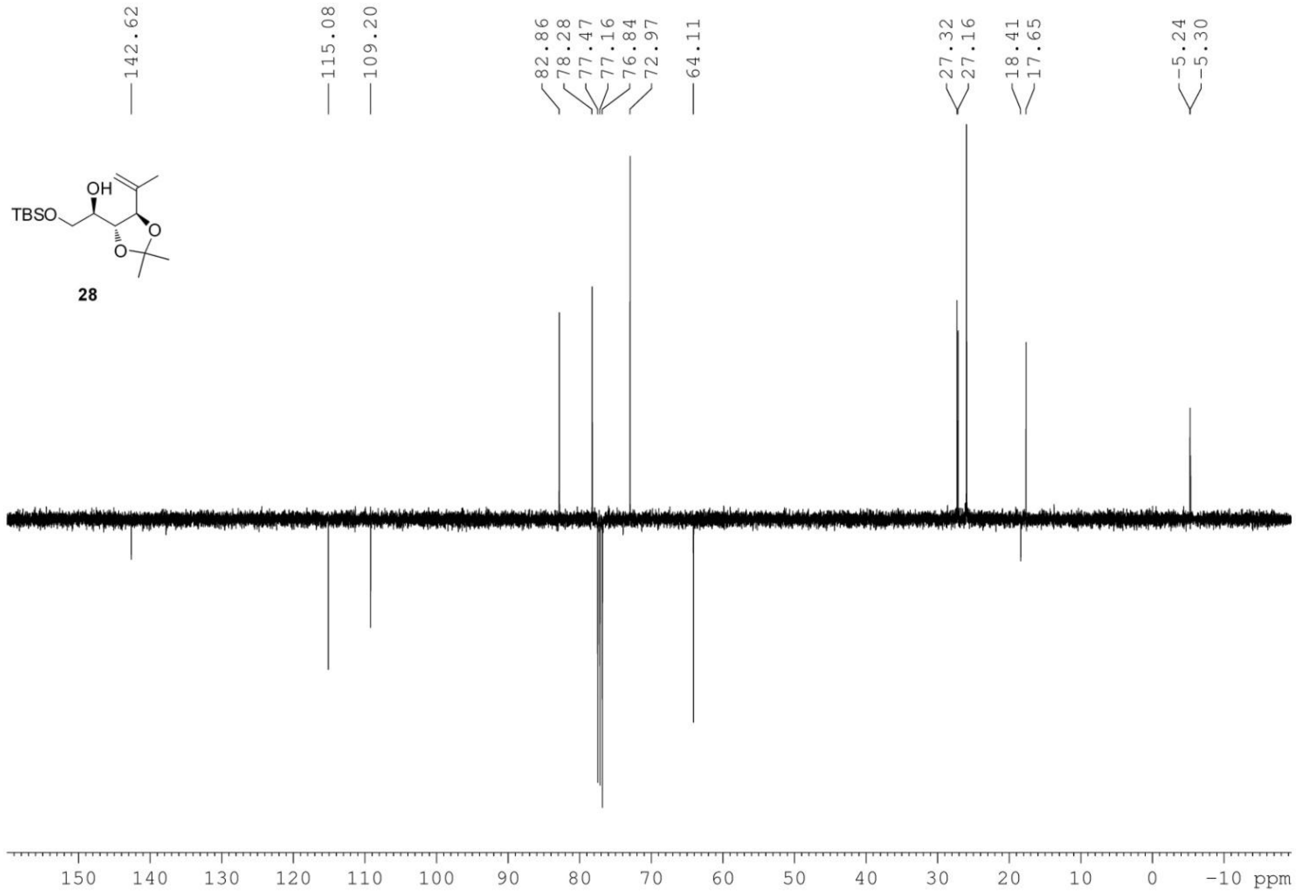
142.62
115.08
109.20
82.86
78.28
77.47
77.16
76.84
72.97
64.11
27.32
27.16
18.41
17.65
-5.24
-5.30
TBSO
OH
O
O
28
150
140
130
120
110
100
90
80
70
60
50
40
30
20
10
0
-10
ppm

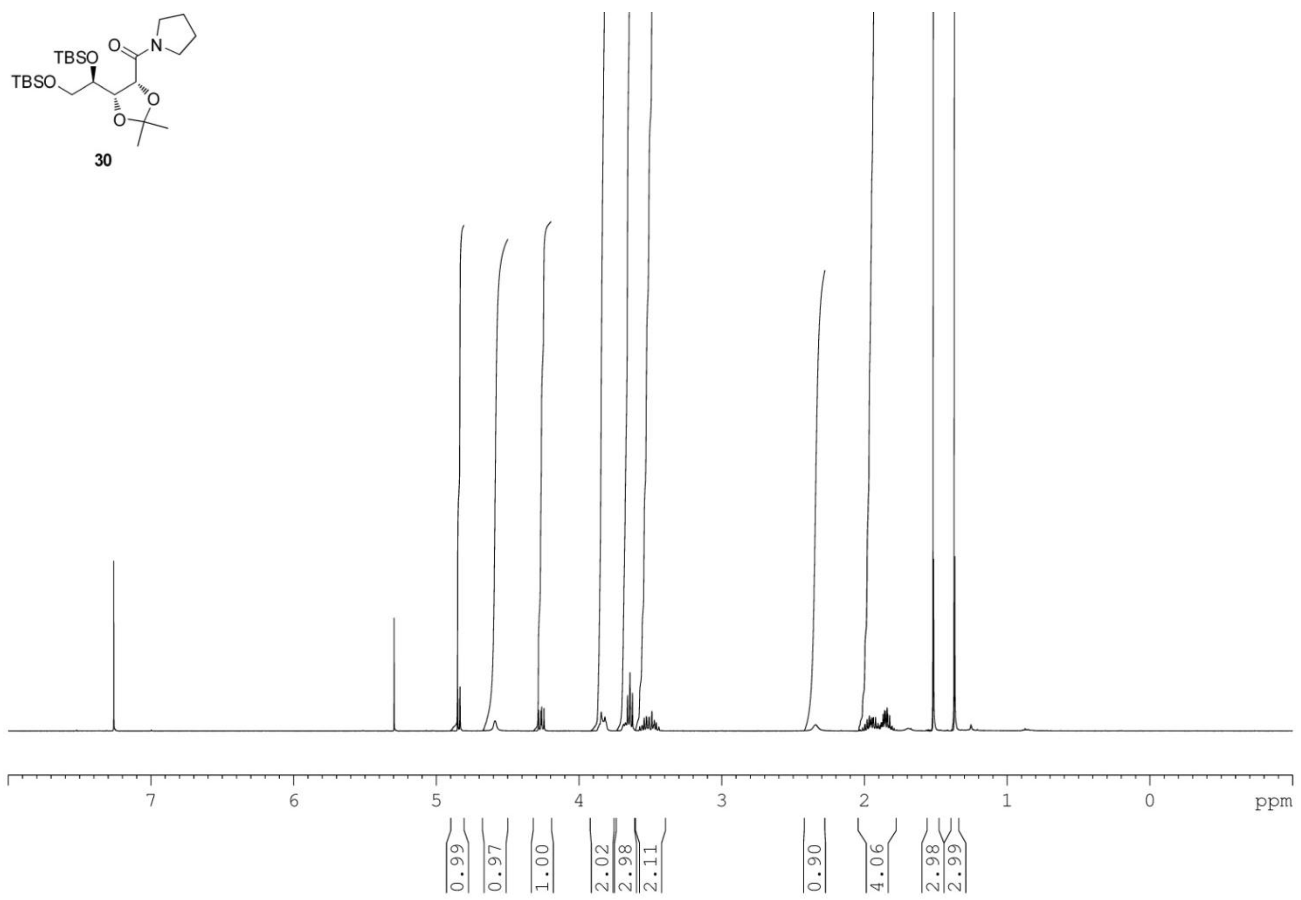
TBSO
TBSO
O
N
O
O
30
7
6
5
4
3
2
1
0
ppm
0.99
0.97
1.00
2.02
2.98
2.11
0.90
4.06
2.98
2.99

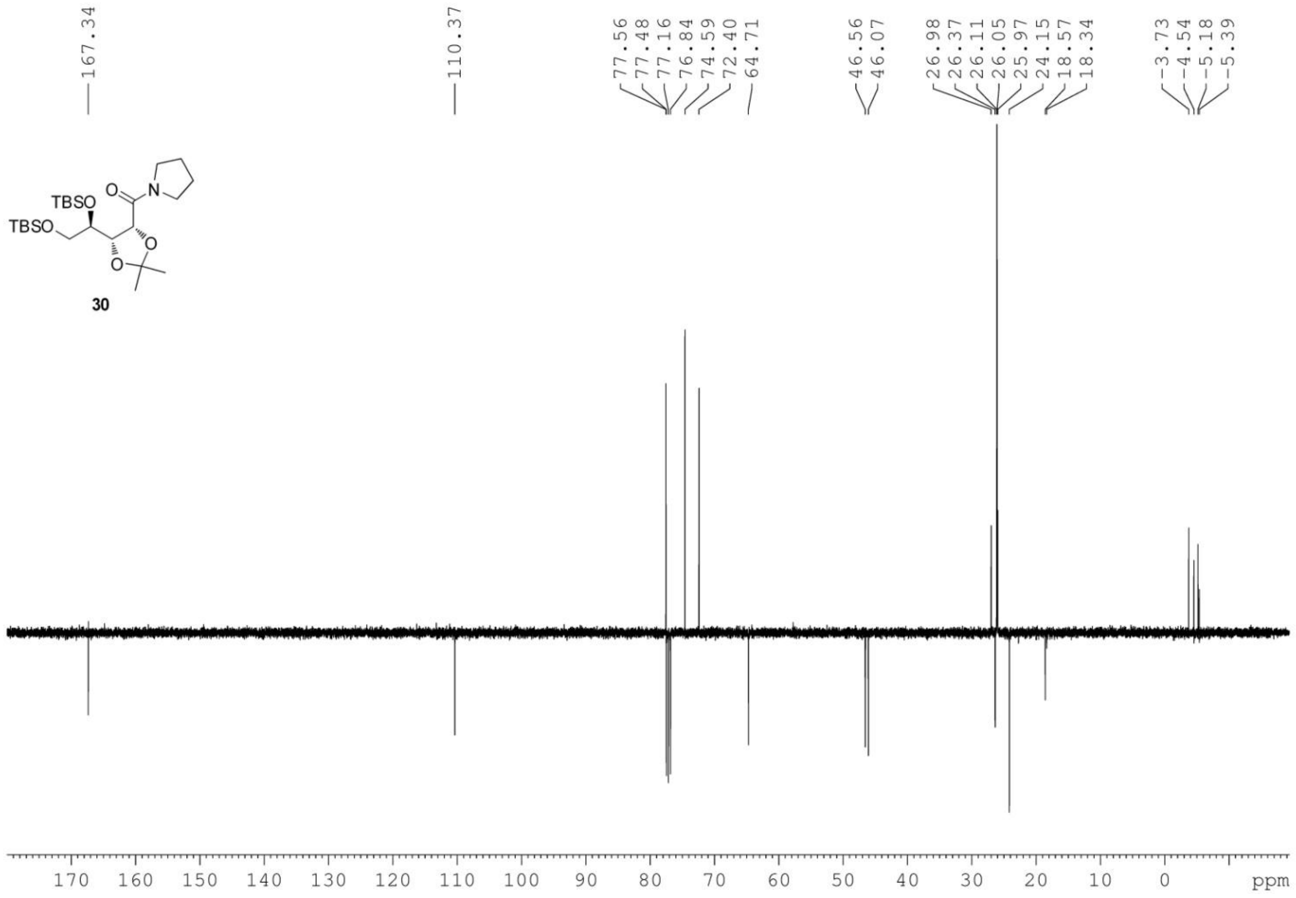
167.34
110.37
77.56
77.48
77.16
76.84
74.59
72.40
64.71
46.56
46.07
26.98
26.37
26.11
26.05
25.97
24.15
18.57
18.34
-3.73
-4.54
-5.18
-5.39
TBSO
TBSO
O
N
O
O
30
170
160
150
140
130
120
110
100
90
80
70
60
50
40
30
20
10
0
ppm

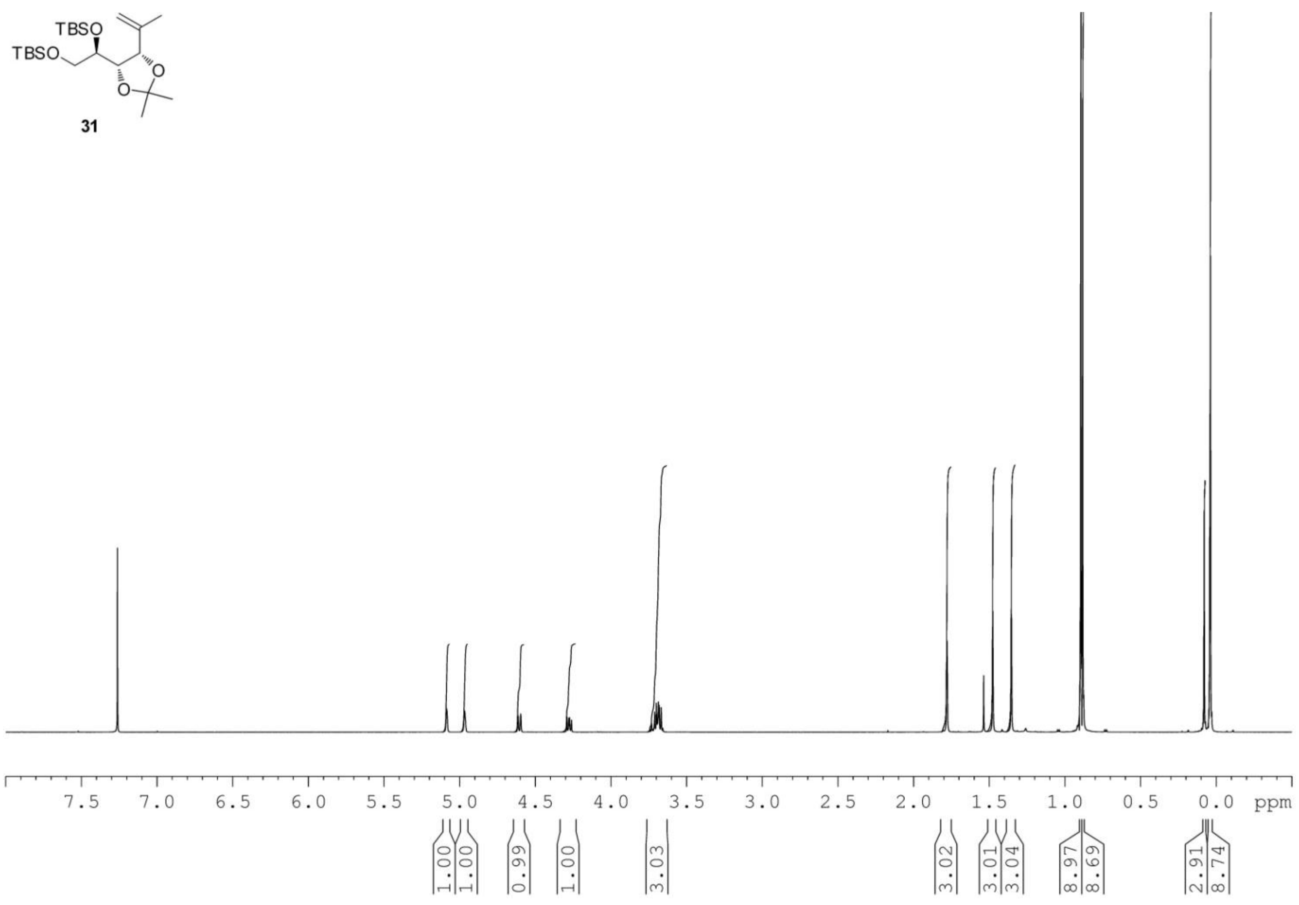
TBSO
TBSO
O
O
31
7.5 7.0 6.5 6.0 5.5 5.0 4.5 4.0 3.5 3.0 2.5 2.0 1.5 1.0 0.5 0.0 ppm
1.00 1.00 0.99 1.00 3.03 3.02 3.01 3.04 8.97 8.69 2.91 8.74

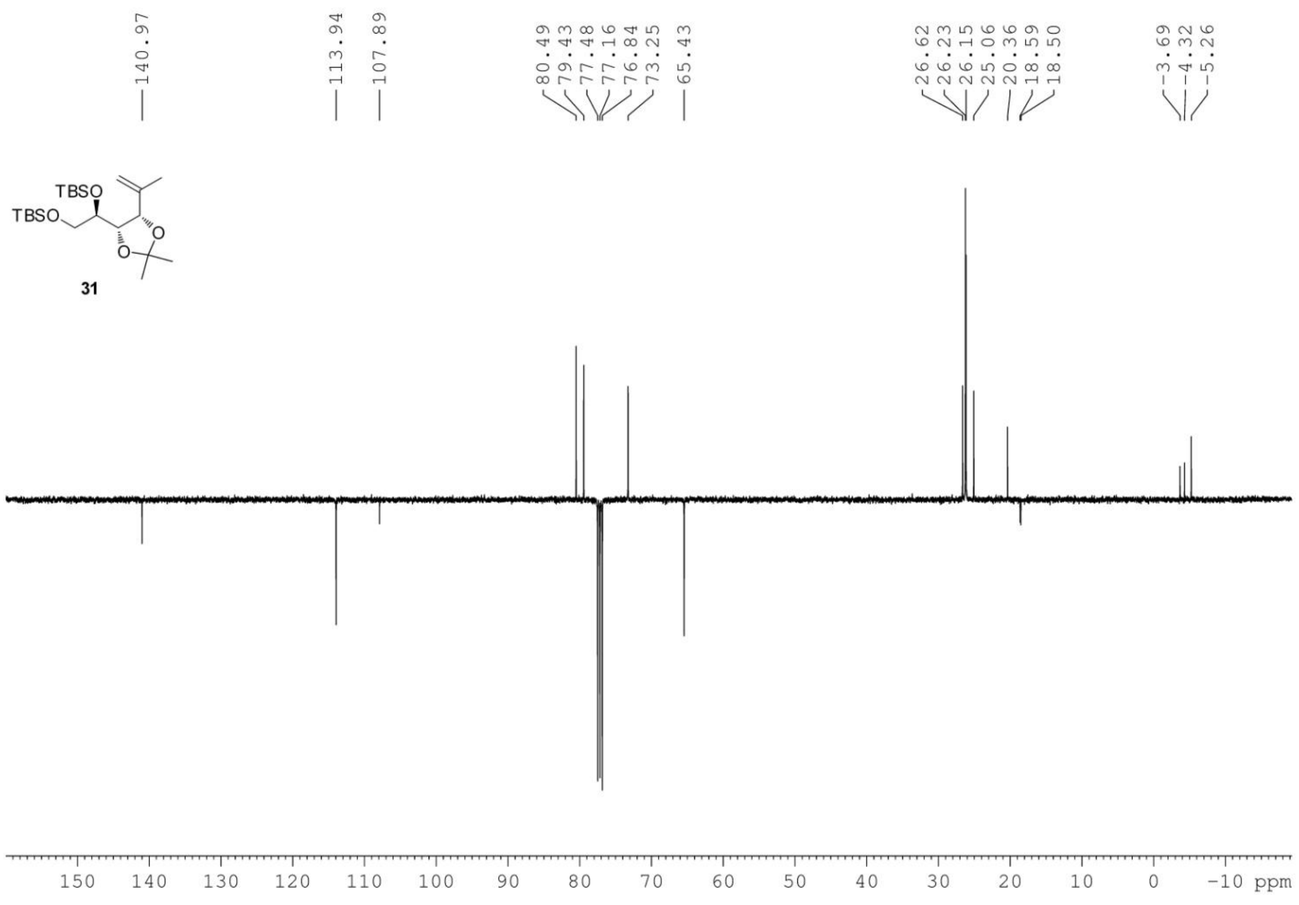
140.97
113.94
107.89
80.49 79.43 77.48 77.16 76.84 73.25
65.43
26.62 26.23 26.15 25.06 20.36 18.59 18.50
-3.69 -4.32 -5.26
TBSO
TBSO
O
O
31
150 140 130 120 110 100 90 80 70 60 50 40 30 20 10 0 -10 ppm

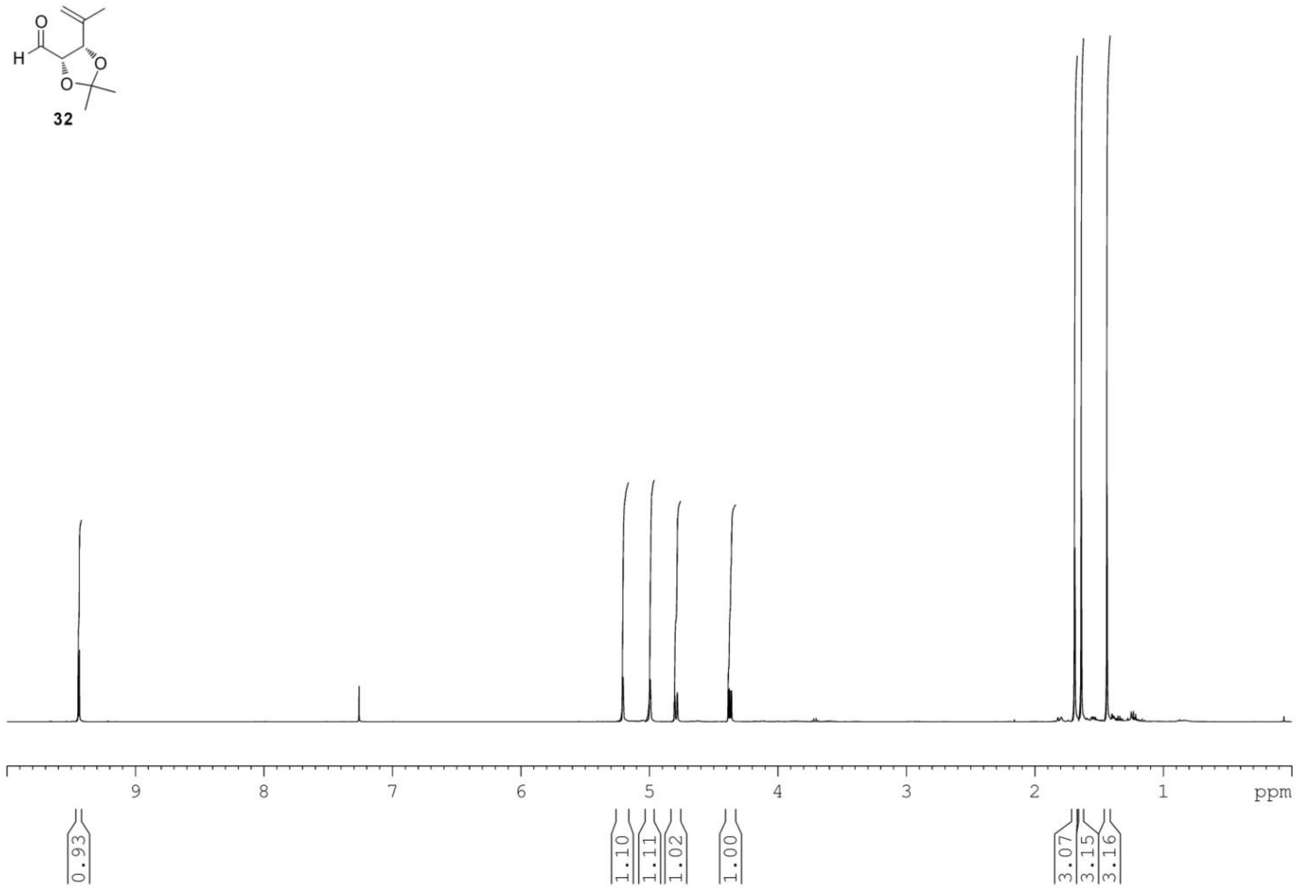

32
9
8
7
6
5
4
3
2
1
ppm
0.93
1.10
1.11
1.02
1.00
3.07
3.15
3.16

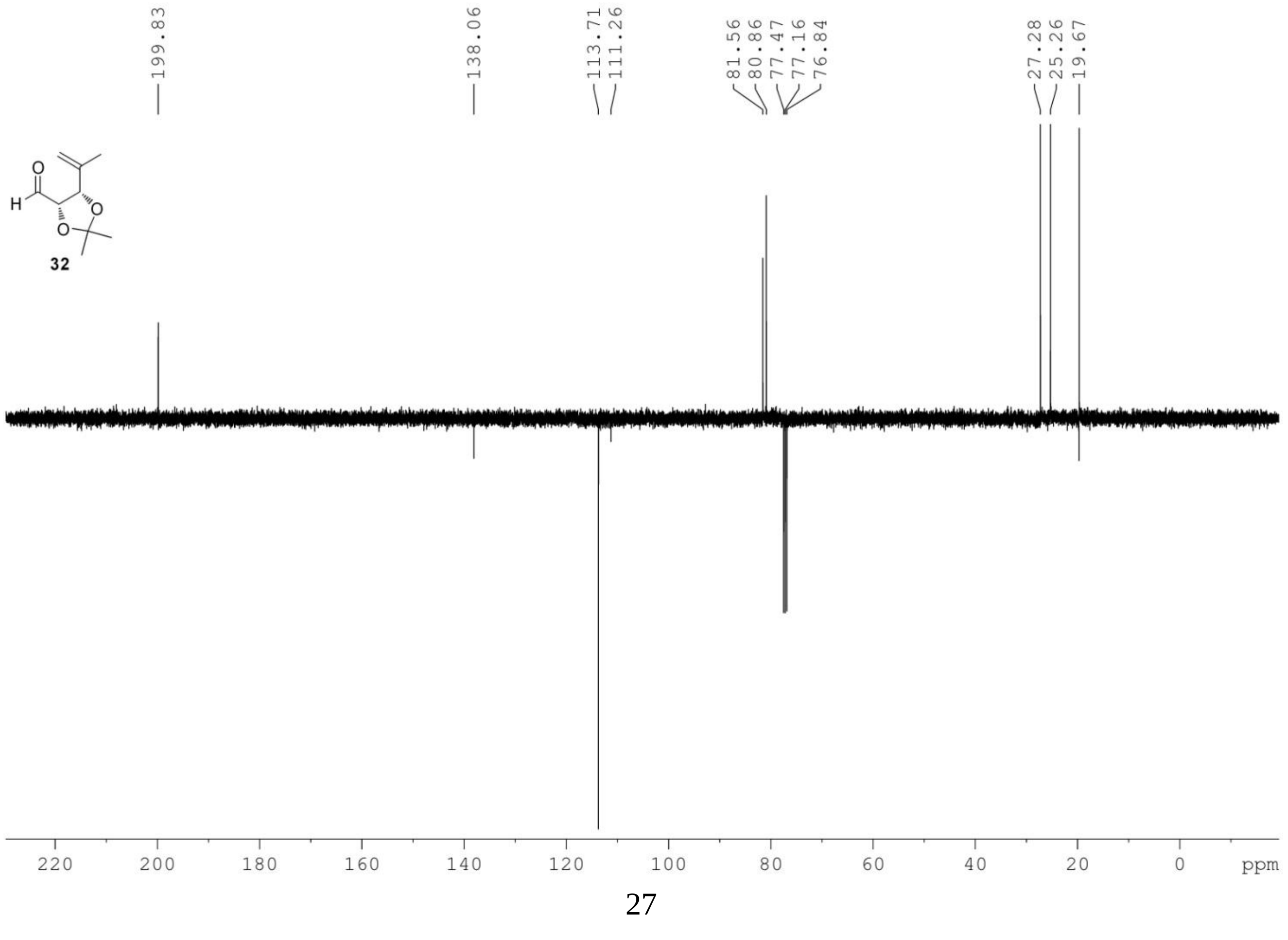

199.83
138.06
113.71
111.26
81.56
80.86
77.47
77.16
76.84
27.28
25.26
19.67
32
220
200
180
160
140
120
100
80
60
40
20
0
ppm

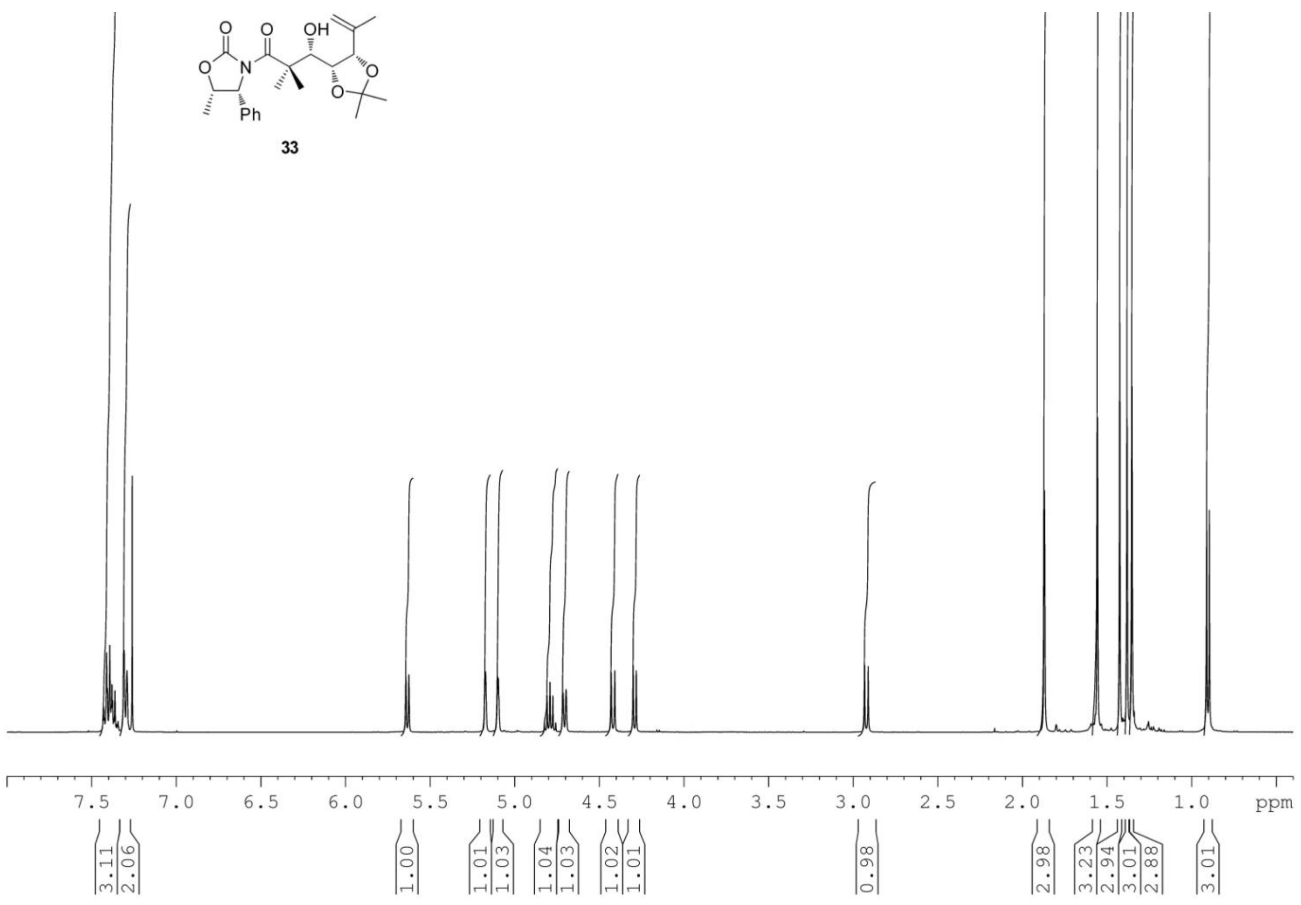
33
7.5 7.0 6.5 6.0 5.5 5.0 4.5 4.0 3.5 3.0 2.5 2.0 1.5 1.0 ppm
3.11 2.06 1.00 1.01 1.03 1.04 1.03 1.02 1.01 0.98 2.98 3.23 2.94 3.01 2.88 3.01

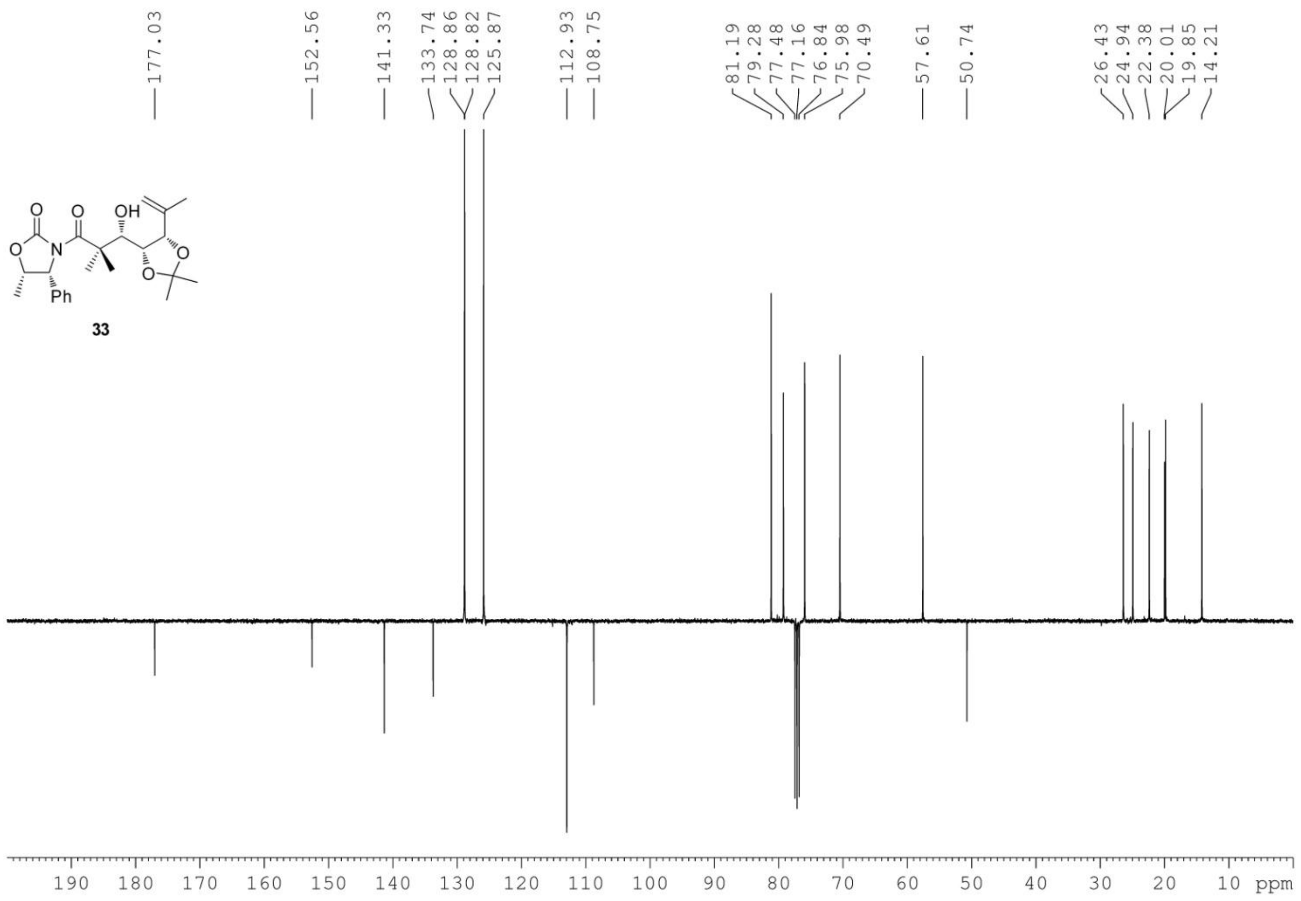
177.03 152.56 141.33 133.74 128.86 128.82 125.87 112.93 108.75 81.19 79.28 77.48 77.16 76.84 75.98 70.49 57.61 50.74 26.43 24.94 22.38 20.01 19.85 14.21
33
190 180 170 160 150 140 130 120 110 100 90 80 70 60 50 40 30 20 10 ppm

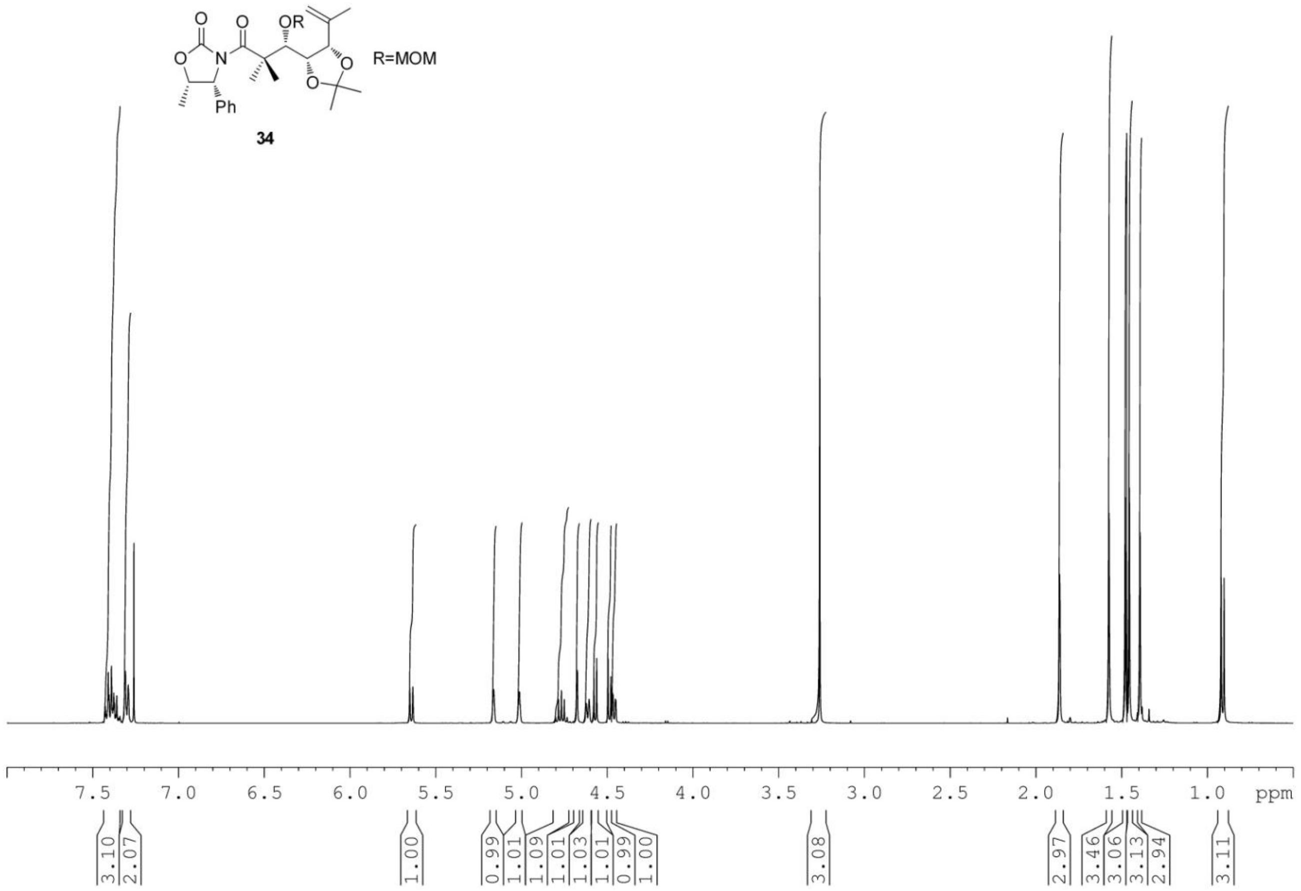
OR
R=MOM
Ph
34
7.5 7.0 6.5 6.0 5.5 5.0 4.5 4.0 3.5 3.0 2.5 2.0 1.5 1.0 ppm
3.10 2.07 1.00 0.99 1.01 1.09 1.01 1.03 1.01 0.99 1.00 3.08 2.97 3.46 3.06 3.13 2.94 3.11

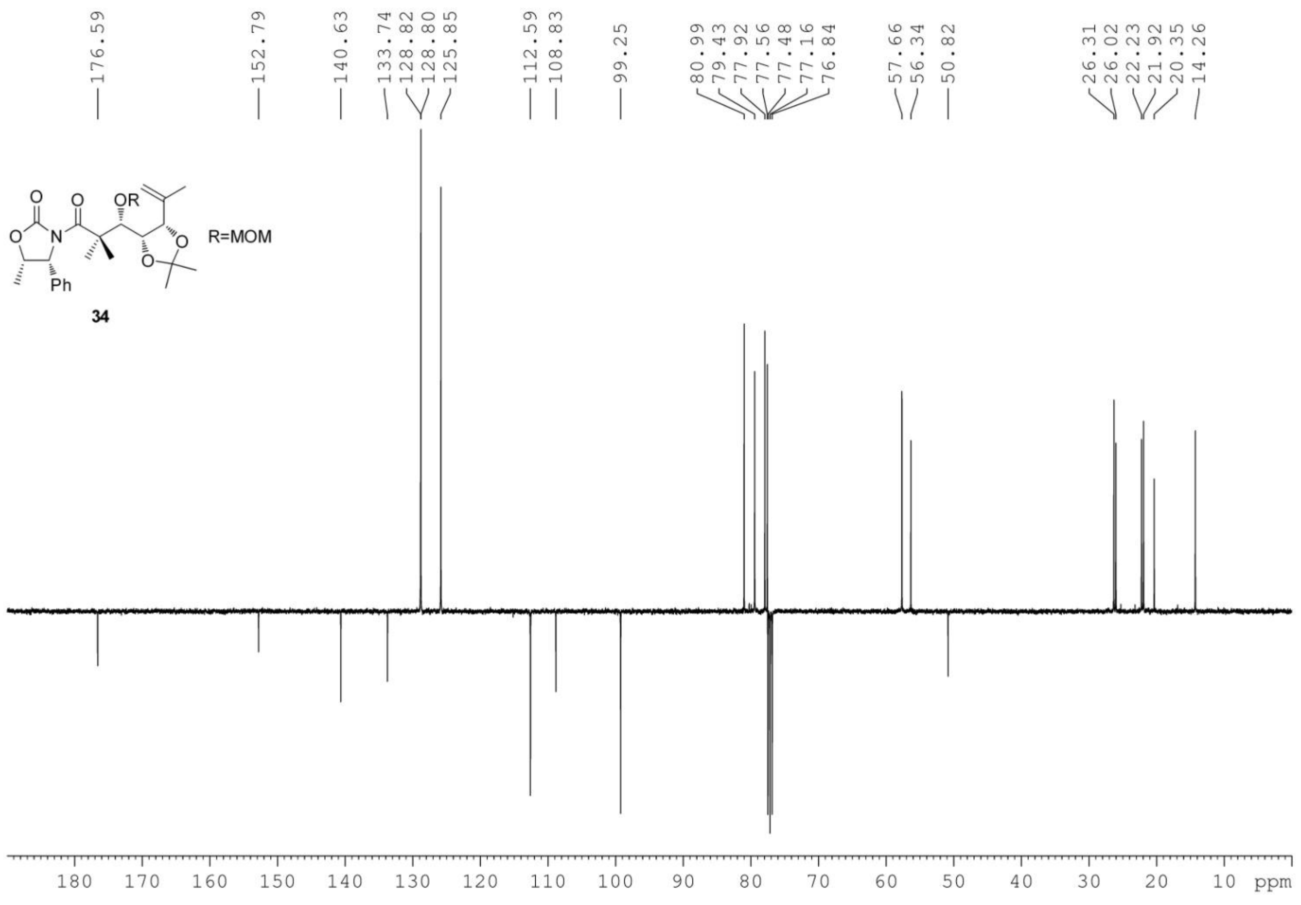
176.59
152.79
140.63
133.74 128.82 128.80 125.85
112.59 108.83
99.25
80.99 79.43 77.92 77.56 77.48 77.16 76.84
57.66 56.34
50.82
26.31 26.02 22.23 21.92 20.35 14.26
OR
R=MOM
Ph
34
180 170 160 150 140 130 120 110 100 90 80 70 60 50 40 30 20 10 ppm
